# Modeling Solvent Effects in Quantum Chemical Calculation of Relative Energies and NMR Chemical Shifts for Azithromycin

**DOI:** 10.1021/acs.jpca.4c08015

**Published:** 2025-02-22

**Authors:** Haroldo C. Da Silva, Isabel S. Hernandes, Wagner B. De Almeida

**Affiliations:** † Laboratório de Química Computacional e Modelagem Molecular (LQC-MM), Departamento de Química Inorgânica, Instituto de Química, Universidade Federal Fluminense (UFF), Outeiro de São João Batista s/n, Campus do Valonguinho, 24020-141 Centro, Niterói, RJ, Brazil; ‡ Departamento de Físico-Química, Instituto de Química, Pavilhão Haroldo Lisboa da Cunha, Universidade do Estado do Rio de Janeiro (UERJ), Rua São Francisco Xavier, 524, 20550-013 Maracanã, Rio de Janeiro, RJ, Brazil

## Abstract

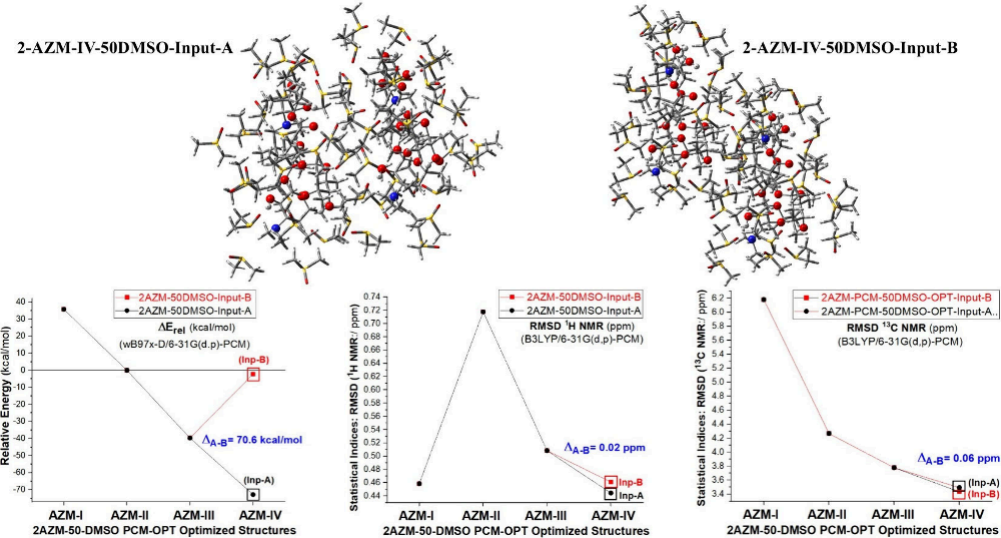

This work presents an investigation into the effect of including explicit solvent molecules in DFT (Density Functional Theory) calculations of relative energies (Δ*E*_rel_) and NMR (Nuclear Magnetic Resonance) chemical shifts for organic molecules in chloroform, water, and dimethyl sulfoxide (DMSO) solution using the PCM (Polarizable Continuum Model) approach. The large and flexible molecule of the antibiotic azithromycin (AZM) containing five OH groups and other polar centers susceptible to interacting with solvent molecules was used here as a working example. An increasing number of explicit solvent molecules was used in the geometry optimization of the supermolecule (*n* = 5, 15, 25, and 50), which accurately reproduces the first solvation shell. We optimized a large AZM trimer (3AZM-75CHCl_3_) structure at the ωB97x-D/6-31G(d,p)-PCM level, containing 747 atoms, which may roughly simulate a 0.1 M dilute solution, with a good agreement with experimental NMR data. The supermolecule approach offers a robust description of solute–solvent intermolecular interactions, effectively accounting for both short- and long-range effects, making it a reliable method for selecting the predominant conformer in solution. While the effect of including explicit solvent molecules on the DFT calculation of Δ*E*_rel_ and ^1^H NMR chemical shifts (OH protons) is remarkable, it is only moderate for the evaluation of ^13^C NMR spectra, providing support for the use of the continuum solvation model in this case. For highly solvated structures, a degree of arbitrariness in the calculation of relative energies is naturally introduced, mainly due to solvent–solvent interaction, causing a strong dependence of total energies on the initial guess structure used in the geometry optimization procedure, with variation in Δ*E*_rel_ around 70 kcal mol^–1^ being predicted, and, therefore, it may not be quite suitable as a criterion to find the predominant conformer in solution. This does not happen with the DFT calculation of ^1^H NMR chemical shifts (RMSD variations less than 0.1 ppm were observed for distinct initial guess structures), which are more strongly influenced by the local chemical environment. This is an interesting result regarding the use of an explicit solvent model in DFT calculations for organic molecules.

## Introduction

The determination of the molecular structure of a chemical compound in solution consists of a key step for understanding the properties of drugs at a molecular level, since the biological environment often solvates them, giving the possibility of changing the conformational structure due to interactions with the solvent. Although the molecules are in constant motion and, therefore, conformational (or structural) changes may dynamically take place as a function of time, it is plausible to conceive that some molecular structures are more likely to be observed than others, having a high Boltzmann statistical weight. The knowledge of these predominant structures in solution is crucial for understanding the interaction with biological targets and, consequently, the mechanism of action of drugs.

When a macroscopic sample in solution is exposed to a magnetic field, the resulting response (measured through chemical shift values) is the domain of nuclear magnetic resonance (NMR) spectroscopy, which is directly related to the predominant molecular structure present at the time of the experiment. This correlation is based on the dependence of the chemical environment of the nuclei on structural changes. The experimentally measured NMR spectrum is strongly influenced by the interaction of the predominant conformation (the “molecular structure”) with the applied magnetic field. Certainly, other structural determination methods, particularly those based on X-ray and electron diffraction, may be much more accurate; however, in solution, NMR remains the most precise technique.

Theoretical modeling of solvent effects is challenging, which makes computational methods essential for addressing this complexity. The computationally more feasible way is using continuum models, for example, the PCM (polarizable continuum model)^1^ and SMD (solvation model based on density).^2^ The word “continuum” denotes that the solvent is not represented explicitly as a collection of discrete solvent molecules but, rather, as a dielectric medium. More specifically, the solvent is approximated as a structureless continuum, whose interaction with the solute is mediated by its permittivity. Therefore, specific solute–solvent interactions are not contemplated in the continuum solvation models, and this can be relevant. Including explicitly molecules is more feasible to carry on using classical simulation methods, for example, Molecular Dynamics (MD).^3^ For quantum chemical calculations, there is no well-defined procedure to make an adequate selection of the number of explicit solvent molecules that should surround the solute (usually, the first solvation shell is required). One possibility is the use of selected frames of MD simulation as a starting point for quantum chemical geometry optimizations since a methodology that considers electrons explicitly is required for theoretical evaluation of NMR chemical shifts. Another possible way to address the solvent effect is to use chemical reasoning to manually place solvent molecules strategically around potential sites for solute–solvent interactions, which has been shown to be an adequate procedure.^4^ Investigating the impact of a chosen solvation model on the quantum chemical calculation of energetic and spectroscopic properties of organic molecules, particularly those relevant to biological applications, is highly valuable. This is the primary focus of this work.

**Scheme 1:**
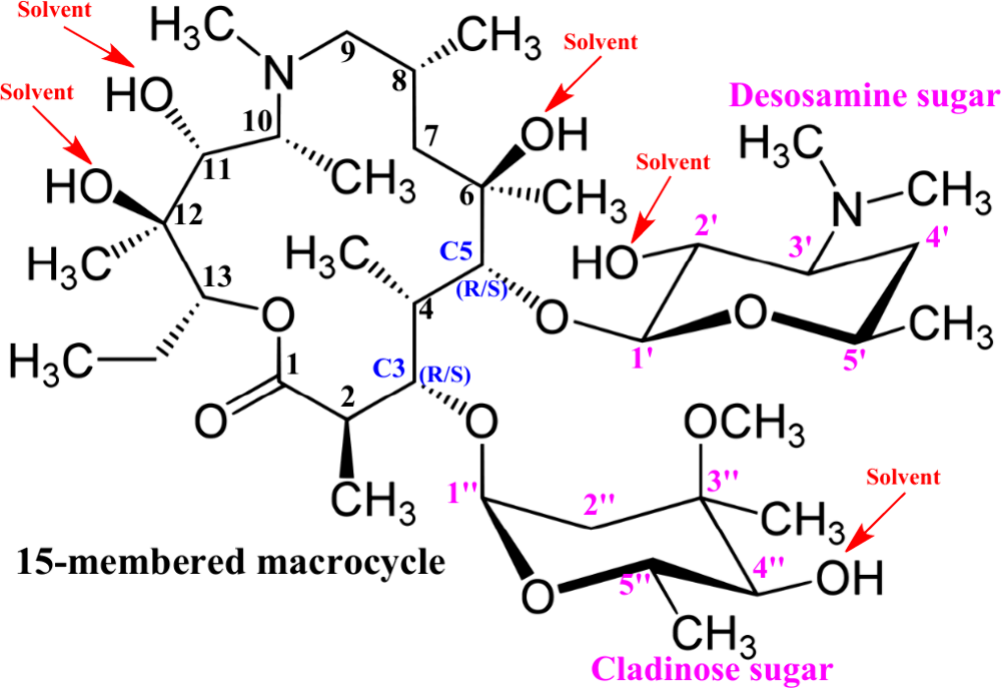
Structural Formula of Azithromycin The numbering scheme and potential sites (OH groups) for interaction with solvent molecules are shown.

In this article, we tackled this problem using as a working example the antibiotic azithromycin (AZM), an antimicrobial medication belonging to the macrolide family used to treat and manage bacterial infections, including community-acquired pneumonia^5^ and sexually transmitted diseases. AZM (Scheme 1) is a large and flexible organic compound containing five OH groups and other polar centers susceptible to interaction with solvent molecules, being a representative molecule for investigating solvent effects. We used the Density Functional Theory (DFT)^6^ as the theoretical methodology, in conjunction with the PCM model,^1^ with the inclusion of explicit solvent molecules, for relative energies and NMR calculations and experimental NMR data (CDCl_3_, D_2_O, and DMSO-*d6* solution) as a reference for comparison with theoretical NMR results. Our theoretical relative energies and NMR chemical shift results allowed us to draw interesting conclusions regarding quantum chemical calculations of molecular properties including solvent effects for the prediction of the predominant structure in solutions.

## Calculations

**Figure 1:**
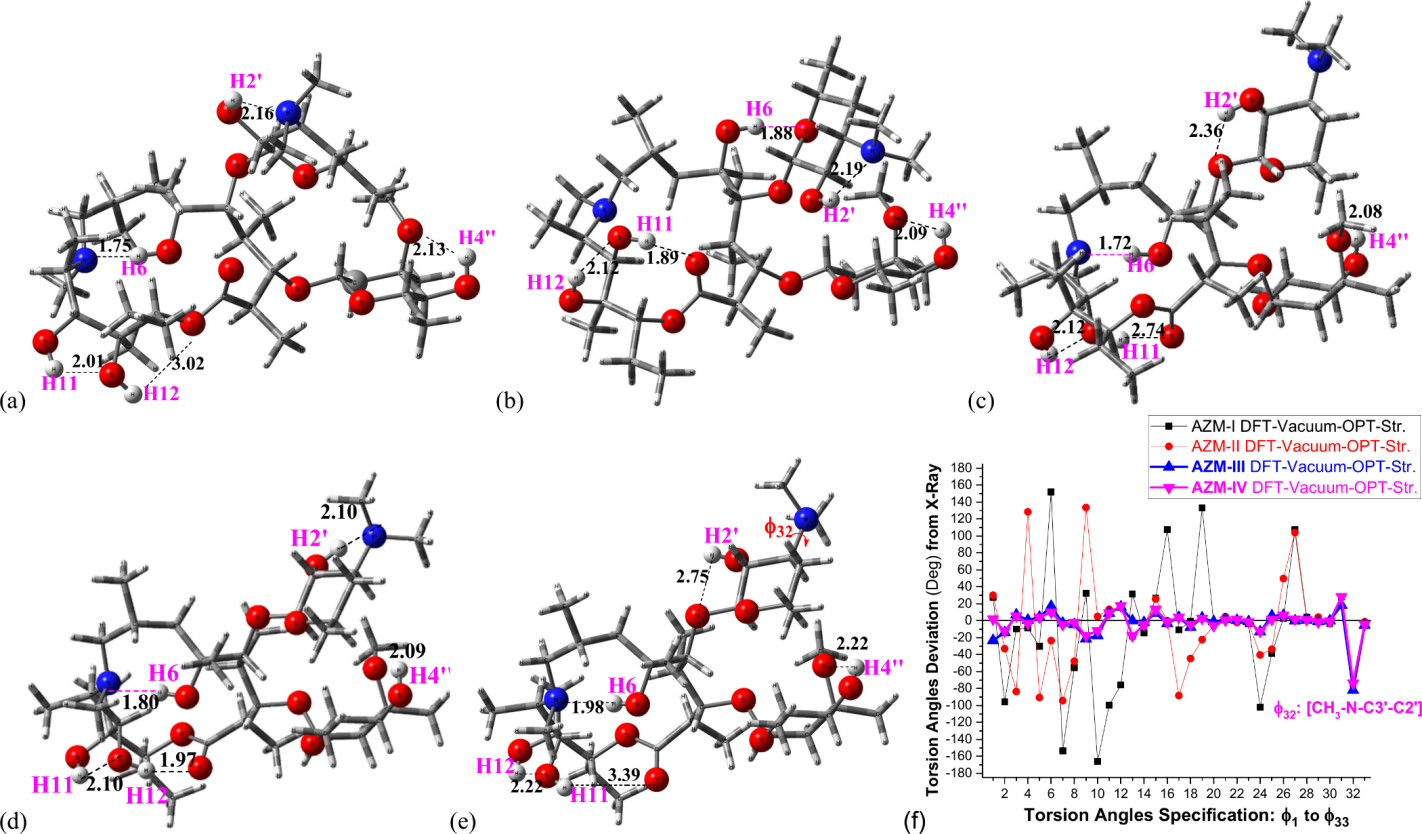
Relevant (a–d) ωB97x-D/6-31G(d,p) fully optimized structures of AZM and (e) X-ray structure. Heteroatoms (N and O) which may be involved in hydrogen bonds are highlighted, and OH protons (H2′, H4″, H6, H11, and H12) are indicated. (f) Torsion angle deviations (deg) from X-ray data are shown with (e) torsion angle **φ**_**32**_ (largest change) specified in H-bonds (Å) are quoted. The definition of torsion angles and the full set of values are given as Supporting Information (Table S1). (a) AZM-I, (b) AZM-II, (c) AZM-III, (d) AZM-IV, (e) X-ray Str. (f) Torsion angle deviations (deg) from X-ray.

Four relevant structures previously found on the DFT potential energy surface (PES) for AZM^7^ (named here **AZM-I**, **AZM-II**, **AZM-III**, and **AZM-IV**) and X-ray structure^8^ are shown in Figure 1. Geometries were fully optimized using the ωB97x-D functional^9^ and 6-31G(d,p) basis set.^10^ Intramolecular H-bonds are highlighted (O–H2′, O–H4″, O–H6, and O–H11 and O–H12 protons). The distinct intramolecular H-bond network led to four different conformations of AZM. Using these four structures from Figure 1 as starting points we performed DFT (ωB97x-D/6-31G(d,p) level) geometry optimization using the PCM model^1,11−14^ and including explicit solvent molecules, named PCM-*n*CHCl_3_, PCM-*n*H_2_O, and PCM-*n*DMSO (*n* = 0, 5, 15, and 25). We simulated a large molecular solvated system containing two AZM molecules and 50 explicit solvent molecules (748 atoms in the case of DMSO solvent, named 2AZM-PCM-50DMSO), optimized at the ωB97x-D/6-31G(d,p)-PCM level, which may roughly represent a dilute solution (0.1 M). To simulate the concentration of the macroscopic solution, we approximated the 2AZM+50 H_2_O molecular dimer system to a cube with an edge equal to the largest interatomic distance in the DFT optimized input (31 Å), resulting in a volume of 2.98 × 10^–26^ m^3^, which corresponds to a “molecular concentration” of 0.11 mol/L. NMR calculations of shielding constants (σ) with chemical shifts (δ) determined on a δ-scale relative to tetramethylsilane (TMS) internal reference was done using the Gauge-Independent Atomic Orbital (GIAO) method^15^ and the B3LYP functional.^16,17^ All calculations were done with the Gaussian G09 package.^18^

## Results and Discussion

The description of the solute–solvent interaction can be complemented by adding nonelectrostatic terms (cavitation, dispersion, and repulsion) contribution to the total energy,^19−21^ which is the PCM implementation in the Gaussian package^18^ used in all DFT-PCM geometry optimizations (PCM-*n*CHCl_3_, PCM-*n*H_2_O, PCM-*n*DMSO, *n* = 0, 5, 15, 25 and 50) carried out in this work. Relative energy results for solvated structures using the SMD^2^ continuum model developed by Truhlar and co-workers, along with PCM results omitting the nonelectrostatic terms (named PCM-default) and energy values evaluated in the vacuum using PCM-optimized geometries, will also be reported for comparison. These results will be relevant for a deep analysis of the solvation effect on relative energies of distinct conformers of AZM.

Defining a microsolvation model, i.e., explicitly including one or more solvent molecules around the solute, is not straightforward for large organic molecules like AZM, due to the numerous possible solute–solvent interactions. Taking selected frames from MD simulation as input for DFT-PCM geometry optimizations may not be quite adequate, since the classical and quantum methodologies are based on distinct grounds; therefore, the DFT-PCM local minima may differ substantially from the MD minimized structures. We used our chemical instinct as a strategy to generate initial AZM solvated structures to be used as input for DFT-PCM geometry optimizations, with an increasing number of explicit solvent molecules (from 5 to 25) which we hope reasonably contemplated the first solvation shell. In addition, dimeric solvated AZM structures containing 50 explicit solvent molecules were also optimized at the ωB97x-D/6-31G(d,p)-PCM level.

The simplest solvation model consists of placing five explicit solvent molecules close to each OH hydrogen donor group, as indicated in Scheme 1, which are, in principle, the strongest solute–solvent interactions (named PCM-5CHCl_3_, PCM-5H_2_O, and PCM-5DMSO), according to our empirical knowledge of chemical interaction. The polar OH groups in a given molecule are more likely to undertake intermolecular interactions with partially positive/negative atoms of solvent molecules, based on simple chemical grounds. Our best model of explicit solvation is an AZM dimer structure containing 50 solvent molecules (named 2AZM-PCM-50CHCl_3_, 2AZM-PCM-50H_2_O, and 2AZM-PCM-50DMSO). We will present results using DFT-PCM implicit solvation and including five, 15, 25, and 50 explicit solvent molecules, which we believe are representative solvated systems. We will discuss the results for each solvent (chloroform, water, and DMSO) separately, for reasons of clarity.

At this point it should be mentioned that in a previous study,^7^ we reported an extensive DFT study of plausible structures located on the PES for AZM, through the calculation of relaxed scan potential energy curves varying six relevant torsion angles from 0° to 360°, with a step size of 30°, with the remaining geometrical parameters being optimized, generating 15 guess structures which were subsequently fully optimized at the DFT level of theory. In a second step, aiming to expand the conformation analysis, Molecular Dynamics (MD) simulations were carried out in DMSO and water solvents, providing additional structural information in solution. The last conformations from each simulation were also included in the set of conformers optimized at the DFT level. Therefore, we believe that a comprehensive search for possible AZM structures, encompassing 15 distinct conformers, was carried out and four relevant structures were selected (**M5**, **M12**, **M13**, and **M14**) renamed here as **AZM-I**, **AZM-II**, **AZM-III**, and **AZM-IV**).

Concerning the level of calculation used, ωB97x-D/6-31G(d,p) and B3LYP/6-31G(d,p) for geometry optimization and NMR chemical shift calculations, respectively, it has been shown in a previous paper about inositol isomers^22^ that a comparison of DFT results with *ab initio* MP2 post-Hartree–Fock ^1^H NMR calculations showed that B3LYP functional exhibited the best agreement with experimental data, and it was found very adequate for the prediction of ^1^H NMR chemical shifts. On the other hand, the ωB97x-D functional is well-known for predicting accurate geometrical parameters and relative energies for molecules possessing polar groups where intramolecular hydrogen bond interactions may play an important role. In addition, a more recent paper on nitrogenated compounds^23^ also showed that MP2/6-31G(d,p) and B3LYP/6-31G(d,p) calculated ^1^H NMR deviations from experimental data regarding N–H protons are comparable, with the behavior being similar with the triple-ζ quality 6-311+G(2d,p) basis set. In previous works it has been shown that the B3LYP/6-31G(d,p)-PCM level of calculation is very satisfactory for the prediction of NMR spectra of organic molecules, in general.^24,25^

Regarding the computational procedure to generate solute solvated cluster structures, we used a sequential approach for including explicit solvent molecules in the DFT-PCM geometry optimizations. We started with five solvent molecules placed at each of the five OH groups, using our chemical intuition, as indicated in Scheme 1. Then we used this optimized solvated geometry to generate an input for ωB97x-D/6-31G(d,p)-PCM geometry optimization with 10 solvent molecules, again using our chemical intuition to select the initial position of the new five solvent molecules. A similar procedure was used for including 15, 20, and 25 explicit solvent molecules. We found that this sequential procedure optimizes the computer time, leading to a more efficient protocol. However, we are aware that the final optimized solvated structure will certainly depend on the initial guess input. Optimizing large clusters of solvated solutes is not a straightforward computational task.

### Structural, Energetic, and Spectroscopic Data: Chloroform Solution

The DFT-optimized geometries (in the vacuum) of AZM structures **I**, **II**, **III**, and **IV** used in this work are shown in Figure 1, along with the X-ray structure,^8^ where intramolecular H-bonds involving the five OH groups are indicated. A table containing torsion angle values and the definition of all 32 torsion angles for all four optimized AZM structures and X-ray data are given as Supporting Information (Table S1). It can be seen, from Table S1 and Figure 1f, that structures **AZM-I** and **AZM-II** deviate significantly from the solid-state structure different from structures **AZM-III** and **AZM-IV**, with the torsion angles exhibiting the largest changes specified in Figure 1e. Apart from torsion angle **φ**_**32**_ ([CH_3_–N–C3′,C2′]), which seems more affected by the packing forces in the solid state, a reasonable similarity with X-ray structure is observed for optimized structures **AZM-III** and **AZM**-**IV**, suggesting that the solid-state carbon skeleton is almost maintained in solution.

**Figure 2:**
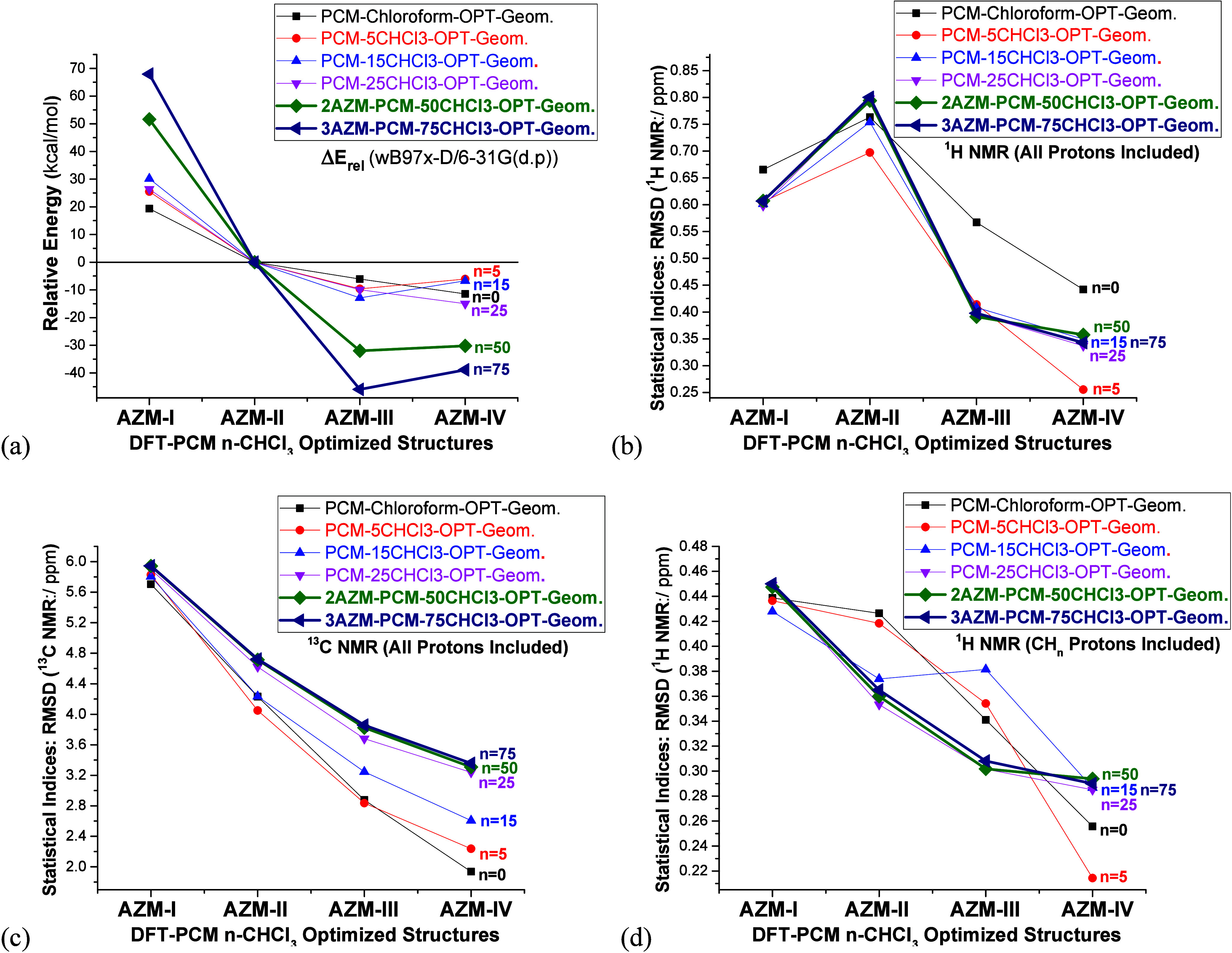
(a) DFT-PCM-Chloroform relative energies (kcal mol^–1^), for structure **AZM-II**, and (b–d) NMR chemical shifts (in ppm) for relevant structures of AZM.

The DFT-PCM-*n*CHCl_3_ (*n* = 0, 15, 25, and 50) results reported in Figure 2 are representative of the explicitly solvated relevant AZM structures **I**, **II**, **III**, and **IV**, shown in Figure 1. Exceptionally for chloroform solvent, AZM trimer solvated structures (3AZM-75CHCl_3_) were also optimized, and the results are included in Figure 2. Experimental NMR data (CDCl_3_) from refs (22, 23, 26, and 27) was used as a reference for the analysis of theoretical NMR chemical shifts. The relative energy trends shown in Figure 2a exhibit an oscillatory behavior, regarding structures **III** and **IV**, with DFT-PCM-*n*CHCl_3_ relative energies (**Δ***E*_**III→IV**_) of −5, +6, +6, −5, +2, and +7 kcal mol^–1^ respectively for *n* = 0, 5, 15, 25, 50, and 75. The energy plot looks smooth; the only difference is the higher stabilization of **AZM-III** and **AZM-IV** structures as the number of explicit solvent molecules increases to 50 (dimer) and 75 (trimer). The stabilization of structures **AZM-III** and **AZM-IV** using 50 and 75 explicit solvent molecules is quite remarkable—around 3–4 times the implicit and PCM-CHCl_3_ model values.

RMSD (root mean square deviation) NMR statistical index data are reported in Figure 2b–d, where the preference for structure **AZM-IV** is quite evident for implicit and PCM-5CHCl_3_ models. The RMSD values were evaluated using eq 1, where Δ*δ*_*i*_ = *δ*_*i*_ – δ_*i*_^exp^ represents the chemical shift (δ) deviation for nucleus *i* relative to its corresponding experimental value, ( is the average of these deviations, and *N* is the total number of nuclei analyzed by NMR.
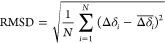
1

The ^13^C NMR RMSD profile is essentially the same for all solvation models, showing the low sensitivity of the ^13^C NMR chemical shifts to the explicit solvation model used in the sense that the same predictions are made. While implicit and PCM-5CHCl_3_ models agree very well, predicting structure **AZM-IV** as the predominant structure in solution (both including and excluding OH protons from the evaluation of RMSD values), extending the number of explicit solvent molecules from 5 to 75 causes a remarkable effect on the ^1^H NMR chemical shift values. Now both **AZM-III** and **AZM-IV** structures may be considered plausible to exist in solution, based on RMSD ^1^H NMR data, having respectively the following values (PCM-50CHCl_3_) of 0.39 and 0.36 ppm (OH protons included) and 0.30 and 0.29 ppm (CH_n_ protons only included). The ^13^C NMR RMSD for **AZM-III** and **AZM-IV** are, respectively, 3.8 and 3.3 ppm. This subject will be addressed later.

**Figure 3:**
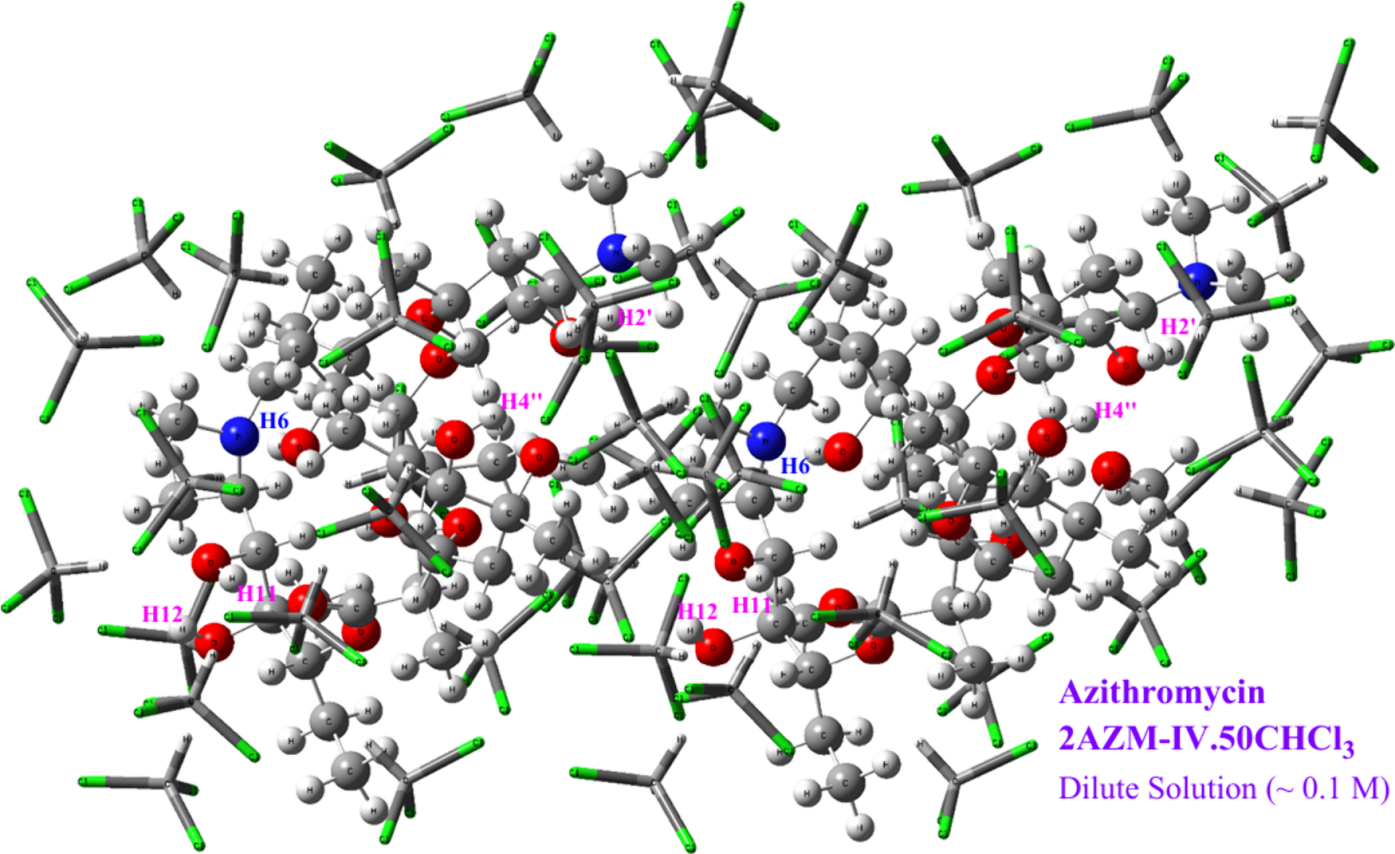
ωB97x-D/6-31G(d,p)-PCM-Chloroform optimized geometry of a dimer of the **AZM-IV** structure in chloroform solution, including 50 explicit CHCl_3_ solvent molecules (named **2AZM-IV-PCM-50CHCl**_**3**_), simulating a 0.1 M solution.

The DFT PCM-50CHCl_3_ optimized structure of the **AZM-IV** dimer is shown in Figure 3. The OH protons susceptible to forming intra- and intermolecular hydrogen bonds are indicated (C6-OH, C2′-OH, C4″OH, C11-OH, and C12-OH). Table 1 contains intramolecular O–H···O and O–H···N distances (in Å) and AZM (OH groups) chloroform shortest solute–solvent intermolecular distances (Å) for dimer (*n* = 50) and trimer (*n* = 75). The corresponding DFT-PCM-*n*DMSO and DFT-PCM-*n*H_2_O (*n* = 0 and 50) values (to be discussed later) are given in parentheses for comparison.

**Table 1:** ωB97x-D/6-31G(d,p)-PCM-*n*CHCl_3_ (*n* = 0 and 50) Intramolecular O–H···O and O–H···N Distances (Å) and AZM(OH groups)–Chloroform Shortest Intermolecular Distances (Å) for **AZM-IV** Structure^a^

AZM Structure IV	C2′–OH···N	C4′′–OH···O	C6-OH···N	C11-OH···O = C	C12-OH···O–H
**AZM-PCM-Chloroform Only**	2.07	2.09	1.78	1.93	2.09
	(2.05)^b^	(2.11)^b^	(1.77)^b^	(1.91)^b^	(2.08)^b^
	(2.05)^c^	(2.11)^c^	(1.77)^c^	(1.92)^c^	(2.08)^c^
**2AZM-PCM-50CHCl**_**3**_	**2.33**	**2.28**	**1.68**	**1.91**	**2.11**
	(3.05)^d^	(2.23)^d^	(1.66)^d^	(2.81)^d^	(2.37)^d^
	(2.47)^e^	(2.21)^e^	(1.63)^e^	(3.74)^e^	(2.16)^e^
**3AZM-PCM-75CHCl**_**3**_	**2.34**	**2.28**	**1.68**	**1.90**	**2.11**

	**AZM···CHCl**_**3**_**Intermolecular Shortest Distance (Å)**				
	**C2′–OH···Cl**	**C4′′–OH···Cl**	**C6-OH···HCCl**_**3**_	**C11-OH···Cl**	**C12-OH···Cl**
**2AZM-PCM-50CHCl**_**3**_	**2.73**	**2.31**	**2.00**	**2.95**	**2.64**
**3AZM-PCM-75CHCl**_**3**_	**2.73**	**2.40**	**1.99**	**2.96**	**2.64**

	**AZM···H**_**2**_**O Intermolecular Shortest Distance (Å)**				
	**C2′–OH···O–H**	**C4′′–OH···O–H**	**C6–O···H–O–H**	**C11-OH···O–H**	**C12-OH···O–H**
**2AZM-PCM-50H**_**2**_**O**	**1.80**	**1.87**	**1.84**	**1.69**	**1.79**

	**AZM···DMSO Intermolecular Shortest Distance (Å)**				
	**C2′–OH···O = S**	**C4′′–OH···O = S**	**C6–O···S = O**	**C11-OH···O = S**	**C12-OH···O = S**
**2AZM-PCM-50DMSO**	**1.84**	**1.98**	**3.17**	**1.72**	**1.97**

a The corresponding DFT-PCM-*n*H_2_O values are given in parentheses.

b ωB97x-D/6-31G(d,p): AZM-IV-PCM-Water.

c ωB97x-D/6-31G(d,p): AZM-IV-PCM-DMSO.

d ωB97x-D/6-31G(d,p): 2AZM-IV-PCM-50H_2_O.

e ωB97x-D/6-31G(d,p): 2AZM-IV-PCM-50DMSO.

The enhancement of the intramolecular H-bond due to interaction with the solvent is much more pronounced for the DMSO and water polar solvents. On the other hand, the intermolecular solute–solvent distances are longer for the low polar chloroform solvent than water and DMSO. The magnitude of solute-solvation interaction may be measured by the intermolecular distance values, which increase in the order of the solvent polarity: water, DMSO, chloroform. This can be expected to play a role in the relative energies of distinct conformers of AZM, as will be shown later.

Intramolecular H-bonds are sequentially enlarged due to the presence of explicit solvent molecules, except for the C6-OH···N hydrogen bond which is unperturbed due to solute–solvent interaction. In general, the solute–solvent shortest distances tend to be longer for highly solvated models but not necessarily for all OH protons. We conclude that it may result from cooperative intermolecular interactions when the number of explicit solvent molecules increases, changing the H-bond network, relative energies, and NMR chemical shifts. For large solvated systems, there will be an increasing number of solvent–solvent interactions, which can have a great effect on the relative energy of distinct conformers of the same molecule but a much less pronounced effect on the chemical shifts, which are strongly influenced by the local chemical environment.

Seeking the lowest values for statistical indices indicates the best agreement with experimental NMR data (as reported in Figure 2b–d). This is certainly more amenable than analyzing ^1^H NMR spectra, where the best agreement between theoretical and experimental NMR profiles can lead to the determination of the predominant molecular structure in solution. In the case of AZM, there are OH and CH_n_ types of protons, and we may analyze separately the two spectra.

**Figure 4:**
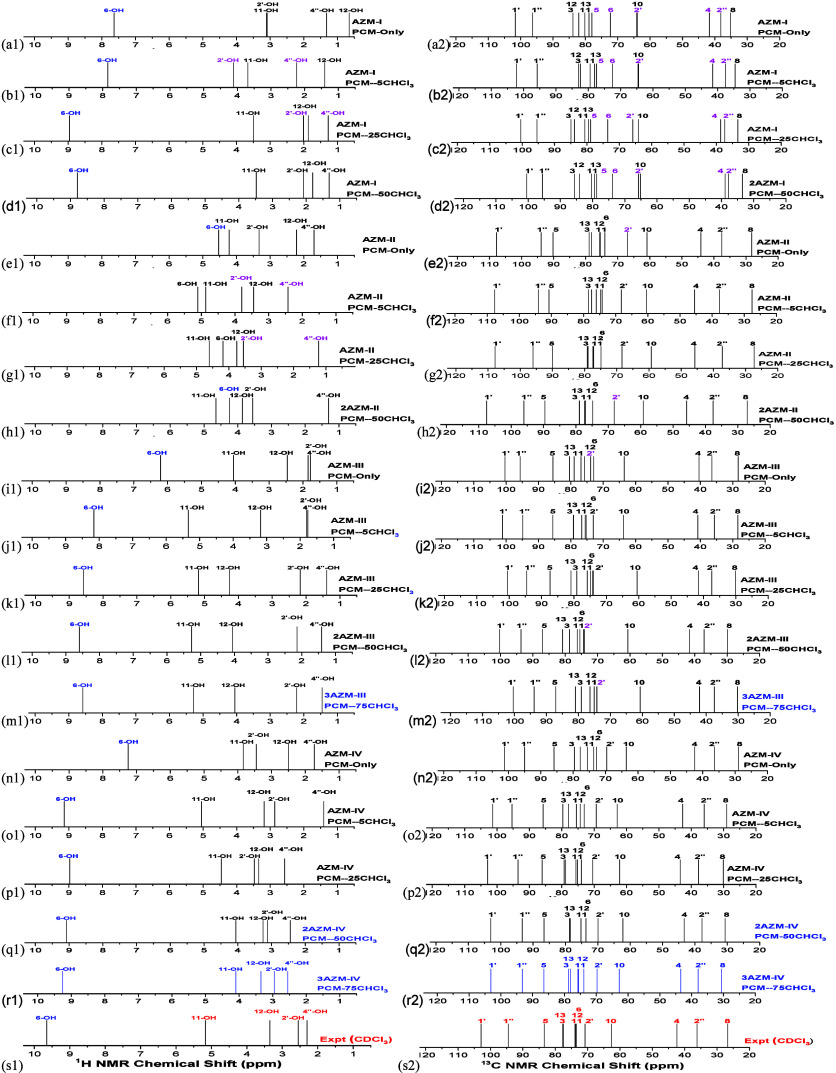
(a1–s1) B3LYP/6-31G(d,p)-PCM-*n*CHCl_3_^1^H (OH protons) and (a2–s2) ^13^C NMR spectra for AZM solvated structures (*n* = 0, 5, 25, 50 and 75).

**Figure 5:**
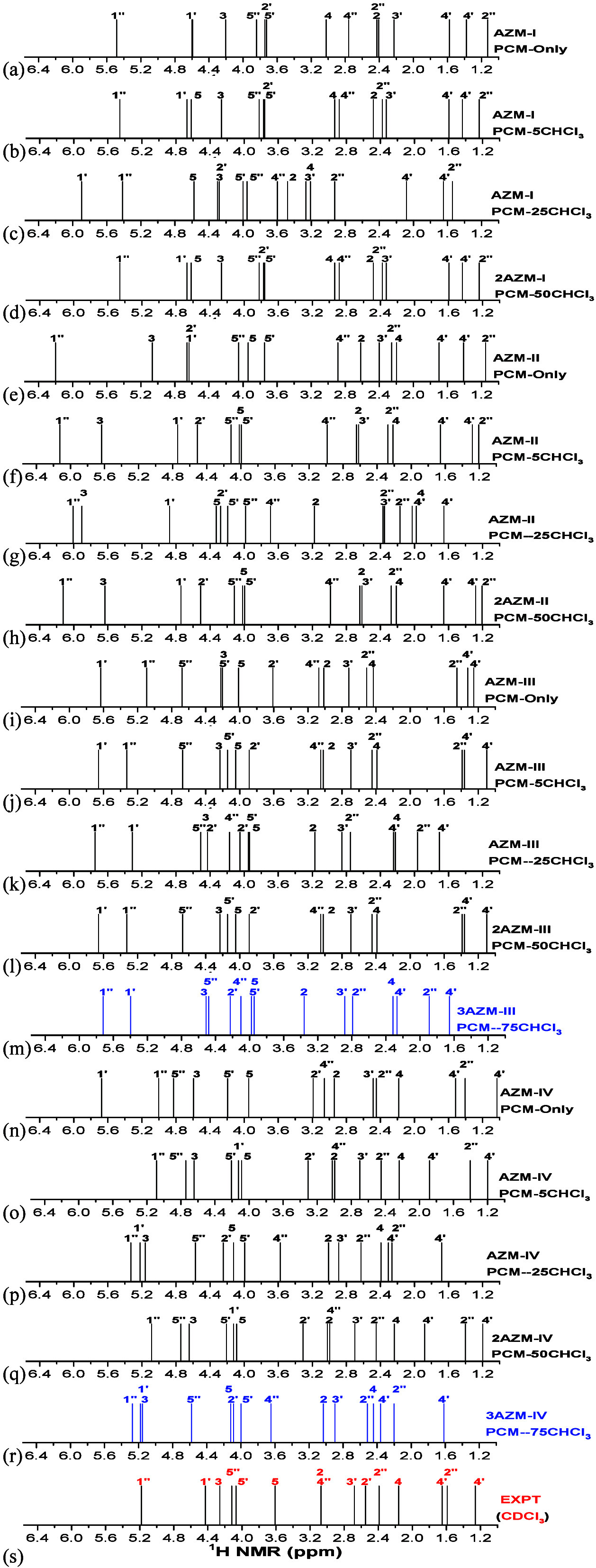
(a–r) B3LYP/6-31G(d,p)-PCM-*n*CHCl_3_ and (s) experimental ^1^H NMR spectra (CH_*n*_ selected protons) for the AZM solvated structures.

Figure 4 shows B3LYP/6-31G(d,p)-PCM-*n*CHCl_3_^1^H NMR (OH protons) and ^13^C NMR spectra for AZM solvated structures (*n* = 0, 5, 25, 50, and 75). NMR spectra for CH_n_ protons are shown in Figure 5 and are too crowded to be analyzed, not bringing a clear indication of agreement with the experimental ^1^H NMR profile. While the effect of including explicit solvent molecules in the calculation of ^13^C NMR chemical shifts is not significant for predicting the NMR profile (Figure 2c), the same is not true for ^1^H NMR spectra. As expected, O–H NMR signals are significantly affected by solvent molecules and in a distinct way by each conformer of the AZM molecule. The same is true for some CH_n_ protons. Seeking the best agreement with the experimental ^1^H NMR profile for the O–H protons is not an easy task. As OH protons are very perturbed by the presence of explicit solvent molecules, relying on the ^1^H NMR OH proton profile for structural determination will always be strongly dependent on the solvation model used, carrying a degree of arbitrariness. Nevertheless, it can be seen from Figure 4-r1 that the solvated trimer of the **AZM-IV** structure shows the best match with the experimental ^1^H NMR profile. Although the ^13^C NMR spectra reported in Figure 4 show an overall uniform pattern, a detailed analysis revealed that some protons marked in pink deviated significantly from the experimental assignments, enabling us to exclude structures **AZM-I** and **AZM-II**, in accordance with the RMSD data (Figure 2c). Such an analysis cannot be made for CH_n_ protons.

Summarizing the analysis in chloroform solution, it can be observed from the results reported in Figure 2 that an oscillatory trend in relative energies is predicted, with a high stabilization of **AZM-III** and **AZM-IV** solvated dimer and trimer structures being observed. Essentially the same trend in RMSD NMR values is predicted, independent of the number of explicit CHCl_3_ solvent molecules used, which may be due to the low polar nature of the chloroform solvent. The low-cost implicit solvation model, combined with the inclusion of just five explicit CHCl_3_ molecules, placed at an adequate position around OH groups, appears sufficient to predict the **AZM-IV** structure as the predominant one in solution according to NMR analysis, consistent with results from other solvation models. As expected, OH and CH_n_ proton chemical shifts are sensitive to the presence of explicit solvent molecules and analysis of the ^1^H NMR profile will be strongly dependent on the number of explicit solvent molecules used, having an intrinsic arbitrariness. Such characteristics are not so explicitly revealed in the RMSD NMR patterns, which seem to be minimized in the evaluation of the statistical indices.

Statistical indices provide an assessment of the average deviation between theoretical and experimental chemical shift data, while analysis of NMR spectra gives us an explicit deviation for each NMR signal, providing a more precise indication of agreement between experimental data and theoretical results, somewhat like fingerprint analysis. However, for large molecules having many protons, as is the case with the AZM molecule, such analysis of the ^1^H NMR profile can be difficult, and the use of statistical indices is recommended. One important aspect of Figure 2 is that increasing the number of explicit CHCl_3_ molecules can affect the relative energy trend but not the RMSD profile. Different from molecular total energy, NMR chemical shifts are not substantially affected by the large number of solvent–solvent interactions, only the nearest solute–solvent type interactions.

### Structural, Energetic, and Spectroscopic Data: DMSO Solution

**Figure 6:**
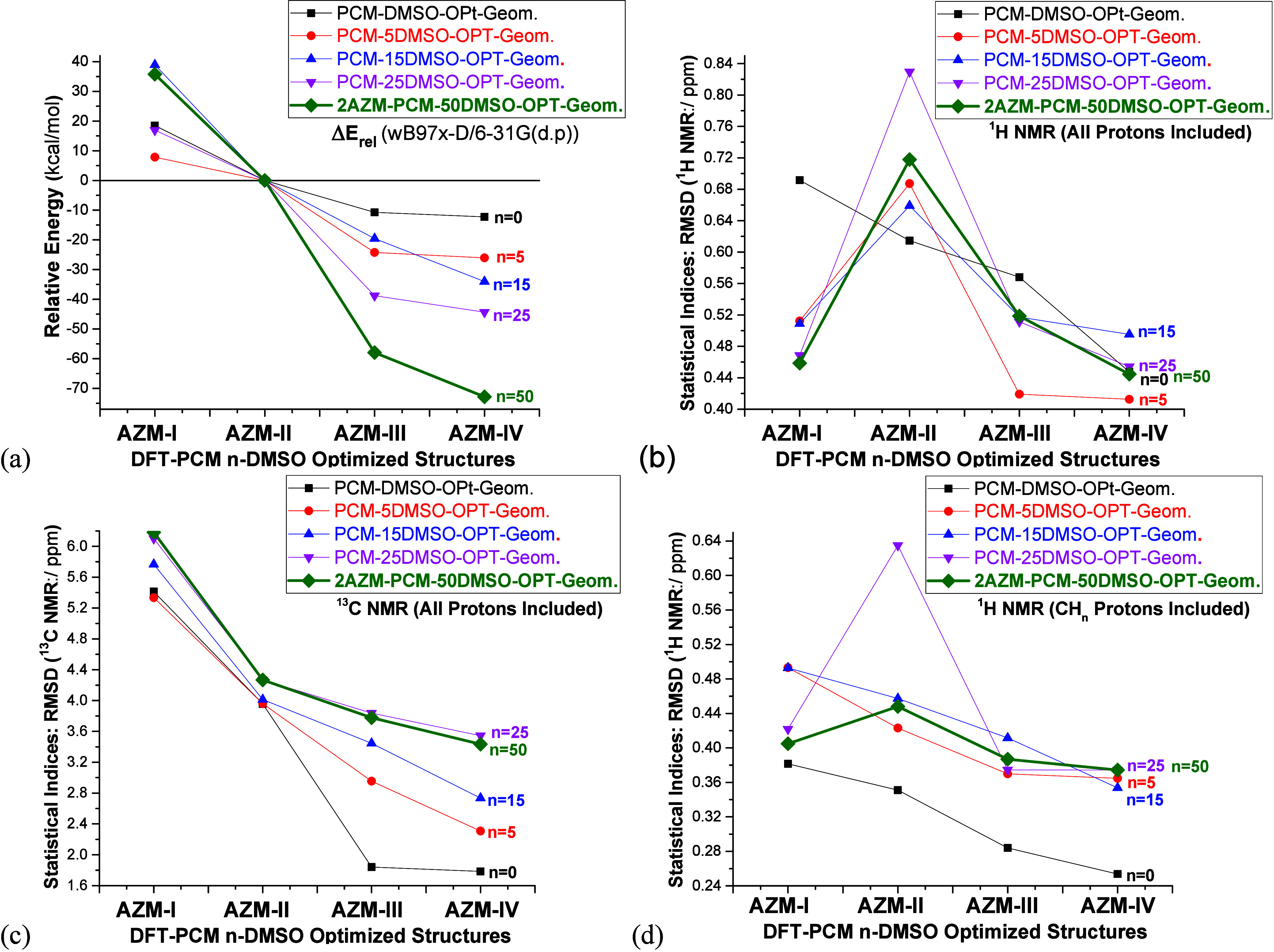
(a) DFT-PCM-DMSO relative energies (kcal mol^–1^), for structure **M12**, and (b, c) NMR chemical shifts (in ppm) for relevant structures of AZM.

Relative energy and NMR statistical indices evaluated in the DMSO solution are listed in Figure 6. Experimental NMR data (DMSO-*d6*) from refs (24 and 28) was used as a reference for the analysis of theoretical NMR chemical shifts. The energy trend calculated including a large number of explicit solvent molecules differs significantly from the implicit and PCM-5DMSO model, with DFT-PCM-*n*CHCl_3_ relative energies (**Δ***E*_**III→IV**_) of −1, −2, −14, −5, and −15 kcal mol^–1^, respectively, for *n* = 0, 5, 15, 25 and 50, being predicted favoring structure **AZM-IV**. For PCM-50DMSO dimeric structures, **AZM-IV** is considerably more stabilized than **AZM-III**, indicating that the energy profile can change substantially for highly solvated structures. Regarding NMR chemical shifts, ^13^C NMR RMSD shows a smooth trend as the solvation model is improved, consistently indicating that structure **AZM-IV** is the preferred one. The same happens with ^1^H NMR chemical shifts, except for structure **AZM-I**, which has a very low RMSD value, differing by 0.01 ppm from structure **AZM-IV** at the PCM-*n*DMSO level (*n* = 15, 25 and 50), perhaps a result of a favorable strong explicit solvent effect on the ^1^H NMR chemical shifts. According to Figure 6, the effect of improving the solvation model for the calculation of ^13^C NMR chemical shifts is much less pronounced than relative energies and ^1^H NMR, in the sense that almost the same RMSD ^13^C NMR trend is obtained using 5, 15, 25, or 50 explicit solvent molecules.

Experimental and calculated NMR spectra, including explicit solvent molecules, are reported as Supporting Information (Figures S1 and S2). The explicit solvent effect on ^13^C NMR spectra (Figure S1) can practically be ignored compared to the ^1^H NMR. As could be expected, the OH proton’s chemical shifts are substantially influenced by solvent effects, and there is no regular pattern for all protons, since the change in the chemical shift will depend on the spatial orientation of explicit solvent molecules close to an OH group. This behavior can be observed in Figure S1, where the solvent effect is distinct for different AZM structures. A comparison between the PCM-5DMSO and PCM-50DMSO structures reveals that the solvent effect cannot be predicted a priori. Each solvation model (containing a number of explicit solvent molecules) may result in a different ^1^H NMR pattern for OH protons.

^1^H NMR spectra for CH_n_ protons are shown in Figure S2. Although the RMSD pattern for CH_n_ protons is quite similar for implicit and PCM-5DMSO solvent models, the ^1^H NMR profiles exhibited significant changes. The same is true when we compare PCM-5DMSO and PCM-50DMSO spectra. For example, the corresponding ^1^H NMR profiles for structure **AZM-II** are very dissimilar. When a comparison with the experimental ^1^H NMR spectrum (in DMSO-*d6*) is made, no clear match is observed for the four AZM DFT-PCM optimized structures. The same holds for the comparison of the OH proton ^1^H NMR spectrum with experimental data. A satisfactory agreement between the average ^13^C NMR profile of structures **AZM-III** and **AZM-IV** with the experimental pattern can be observed from Figures S1-n2 and S1-o2, which is in line with the RMSD curve shown in Figure 6c.

### Structural, Energetic, and Spectroscopic Data: Water Solution

**Figure 7:**
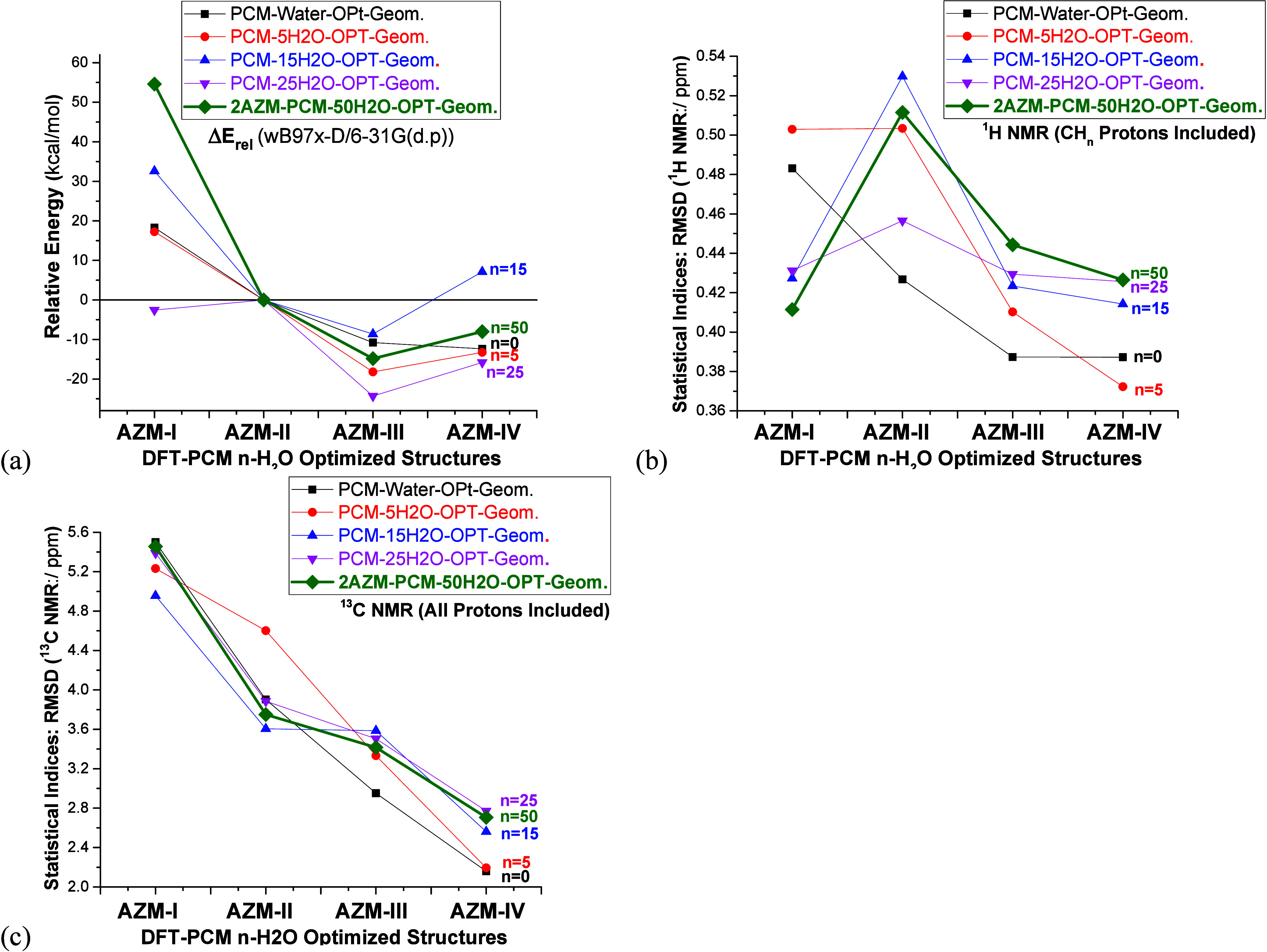
(a) DFT-PCM-Water relative energies (kcal mol^–1^), with respect to structure **M12**, and (b, c) NMR chemical shifts (in ppm) for relevant structures of AZM.

Relative energies and NMR results for the water solvents (DFT-PCM-*n*H_2_O) are shown in Figure 7. Experimental NMR data (D_2_O) from refs (24 and 28) was used as a reference for the analysis of theoretical NMR chemical shifts. The energy profile from Figure 7a, with the inclusion of explicit water solvent molecules, clearly predicted structures **AZM-III** as energetically preferable by more than 5 kcal mol^–1^. When using the polar water solvent, the PCM-5H_2_O solvation model RMSD NMR trend predicted structure **AZM-IV** as being predominant. However, as the number of solvent molecules increases, significant changes in the NMR trend occur due to stronger solute–solvent interactions, with the solvated dimer **AZM-IV** structure again being favored. An interesting result from Figure 7a is how the improved solvation model affects different conformers of the same organic molecule in distinct ways, significantly destabilizing structures **AZM-III** and **AZM-IV**, which, conversely, are highly stabilized in chloroform (PCM-50CHCl_3_) and DMSO solution (PCM-50DMSO), as shown in Figures 2a and 6a, respectively. A change of pattern in the RMSD ^1^H NMR chemical shifts was observed for structure **AZM-I**, similar to the results observed in DMSO (Figure 6b) and different from the behavior in chloroform solution (Figure 2d). However, the ^13^C NMR chemical shifts profile (Figure 7c) follows the same smooth trend predicted for the chloroform and DMSO solvents (Figures 2c and 6c), not changed by improving the solvation model, indicating structure **AZM-IV** as the preferred one.

**Figure 8:**
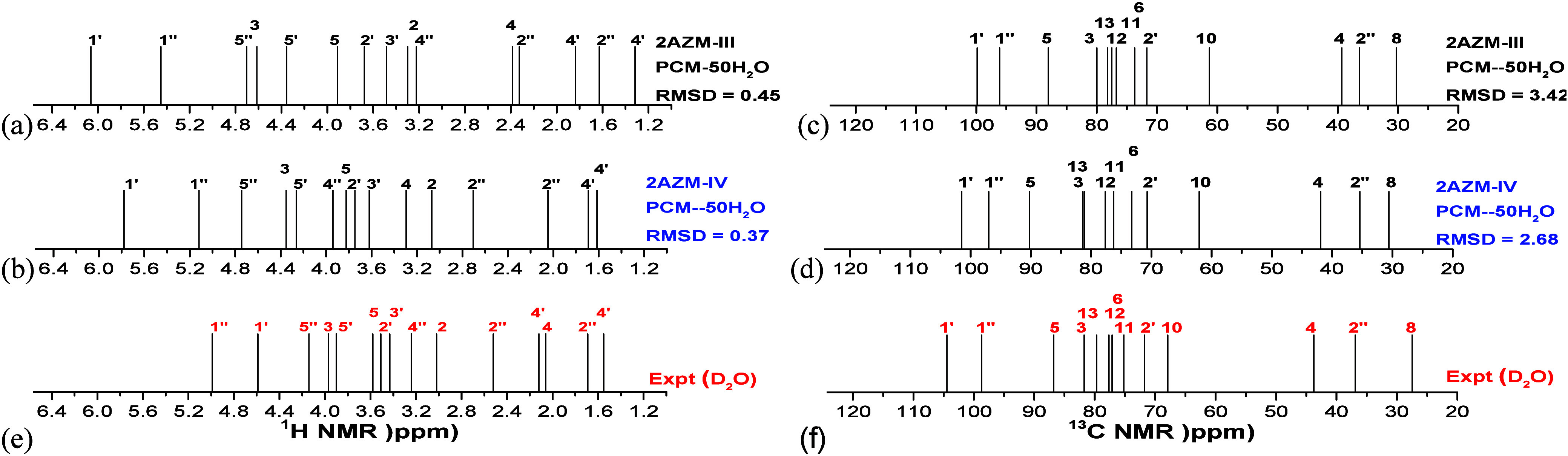
B3LYP/6-31G(d,p)-PCM-50H_2_O (a, b) ^1^H NMR and (c, d) ^13^C NMR spectra for **AZM-III** and **AZM-IV** solvated structures and (e, f) experimental spectrum (in D_2_O). Selected protons were used for easy spectrum visualization.

Experimental and calculated NMR spectra, including explicit solvent molecules, are reported as Supporting Information (Figure S3). It can be seen that no significant change in the NMR profiles, which could be used for structural determination through agreement with the experimental profile, was observed. Therefore, the analysis of PCM-*n*H_2_O NMR spectra was not useful as a tool to elucidate the predominant AZM structure in water solution. Just to illustrate, ^1^H and ^13^C NMR spectra for **AZM-III** and **AZM-IV** structures are shown in Figure 8.

**Figure 9:**
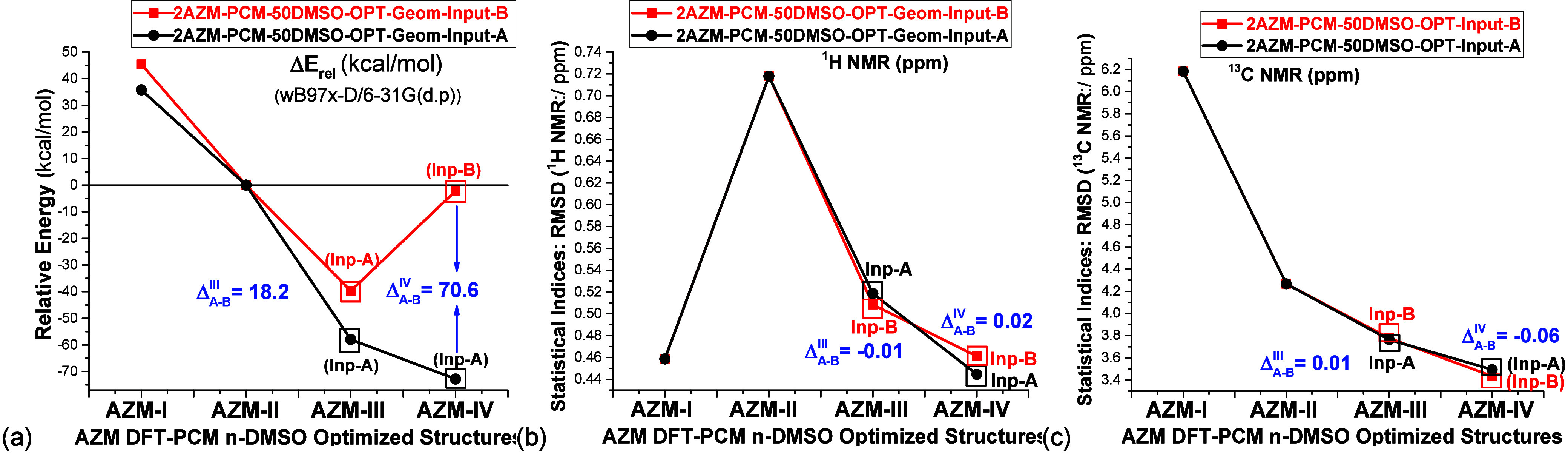
(a) DFT-PCM-50DMSO relative energies (kcal mol^–1^) and (b, c) RMSD NMR values (ppm) for relevant AZM structures, using two distinct manually modified inputs of structures for **AZM-III** and **AZM-IV** in the geometry optimization procedure, named Input-A and Input-B, resulting in different optimized dimeric solvated structures. **Δ**_**A-B**_ is the difference between values calculated for optimized structures **III** and **IV** using, respectively, inputs B and A.

All DFT-PCM optimized structures used to calculate energy and RMSD values reported in Figures 2, 6, and 7 were obtained using an arbitrary guess input geometry named Input-A. We investigated the effect of changing the initial guess input structure in the geometry optimization procedure on the relative energies and NMR chemical shifts for the more plausible azithromycin dimeric structures **2AZM-III** and **2AZM-IV**. This new input was named Input-B and was manually built through inversion of the configuration of one of the AZM units (PCM-25CHCl_3_, PCM-25H_2_O, and PCM-25DMSO), generating a dimer structure quite distinct from that of Input-A, regarding the spatial orientation of the explicit solvent molecules around the solute. The dependence of relative values on the initial guess structure in DMSO solution is remarkable, with the **2AZM-IV-50DMSO** Input-B structure being destabilized by approximately 70 kcal mol^–1^ compared to Input-A, which represents a very large energy difference (Figure 9). Regarding RMSD values, virtually the same results are obtained with both Input-A and Input-B, with maximum deviations (Δ_*A-B*_^III^ and (Δ_*A-B*_^IV^) of 0.02 and 0.06 ppm observed respectively for ^1^H and ^13^C NMR chemical shifts, showing a very low sensitivity to the initial solvated guess geometry.

The results reported in Figure 9 provide a good example of the high sensitivity of relative energies to the initial input structure used in the DFT geometry optimization procedure, totally contrasting with the behavior of the RMSD NMR data. Changing the guest input structures can have a remarkable effect on the relative energy pattern of various conformers of the same molecule, and so an inevitable arbitrariness is introduced, making relative energies for highly solvated molecular systems not trustable. In other words, the solvated structure cannot be uniquely defined. This may be attributed to solvent–solvent interactions, which can affect significantly the total energy of the optimized structures, and, consequently, relative energies, but not the NMR chemical shifts which depend strongly on the local chemical environment, not long-range solute–solvent and solvent–solvent interactions. Therefore, analysis of DFT-calculated NMR chemical shifts seems more adequate than relative energies of various conformers of the same explicitly solvated organic molecule, in which structural elucidation is concerned.

**Figure 10:**
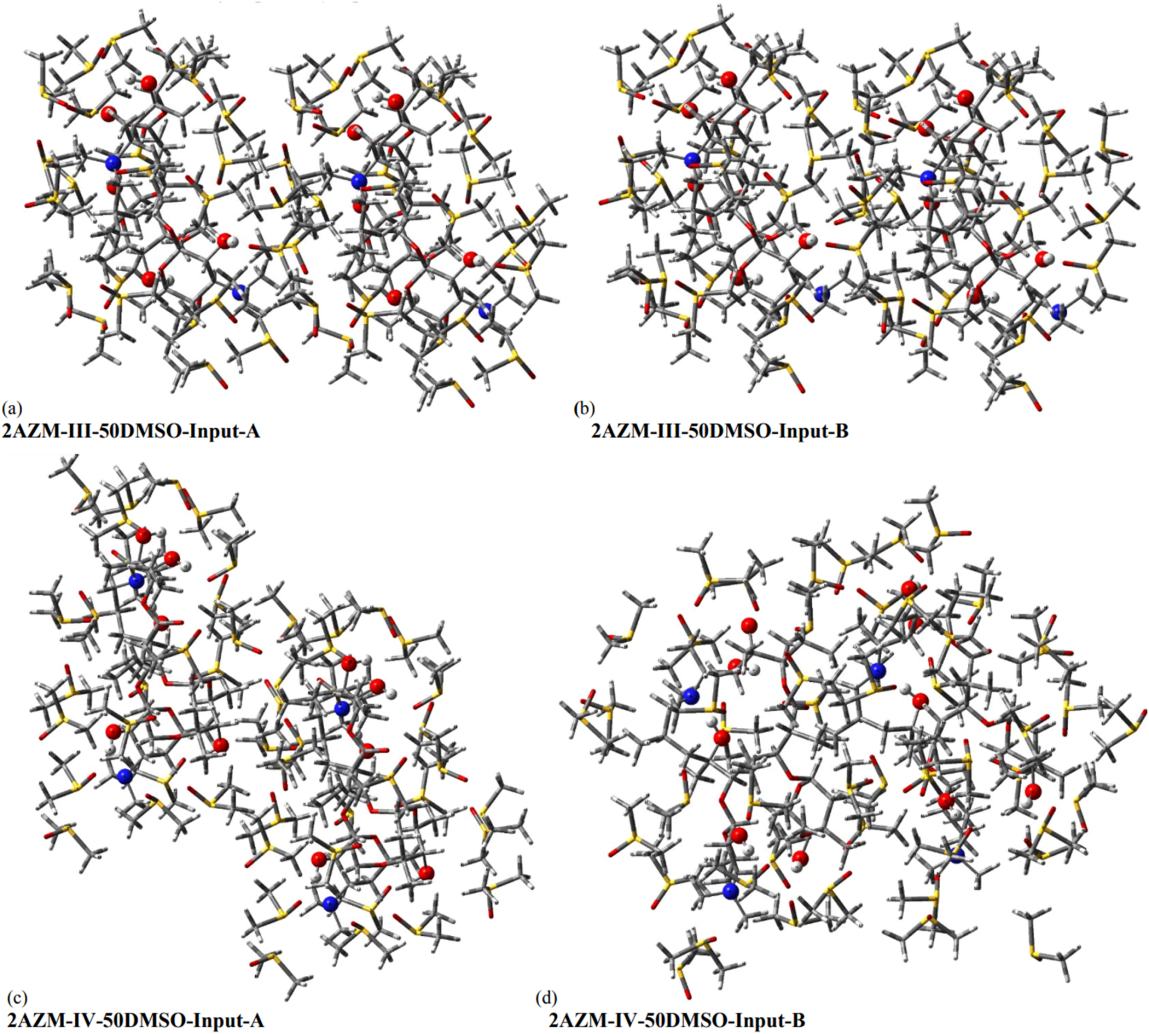
DFT-PCM-50DMSO relative energies (kcal mol^–1^) and RMSD NMR values (ppm) for relevant AZM structures, using two distinct manually modified inputs of structures for (a, b) **AZM-III** and (c, d) **AZM-IV** in the geometry optimization procedure, named **Input-A** and **Input-B**, resulting in different optimized dimeric solvated structures. Nitrogen atoms (blue color) and OH groups (read color) were highlighted for easy visualization.

The four DFT-PCM-50DMSO **AZM-III** and **AZM-IV** structures (Input-A and Input-B), whose energy and RMSD values are reported in Figure 9, are shown in Figure 10, where the structural dissimilarities of Input-A and Input-B optimized structures can be promptly seen. Looking at the AZM solvated dimeric structures from Figure 10 leads us to think that many different guess structures are possible, and so several DFT-PCM-50DMSO optimized dimeric structures can be obtained. Therefore, finding the global minimum energy structure on the potential energy hypersurface becomes an intangible computational task. So, the use of relative energy values as a criterion to elucidate the preferred molecular structure of organic molecules in solution may be questioned.

**Figure 11:**
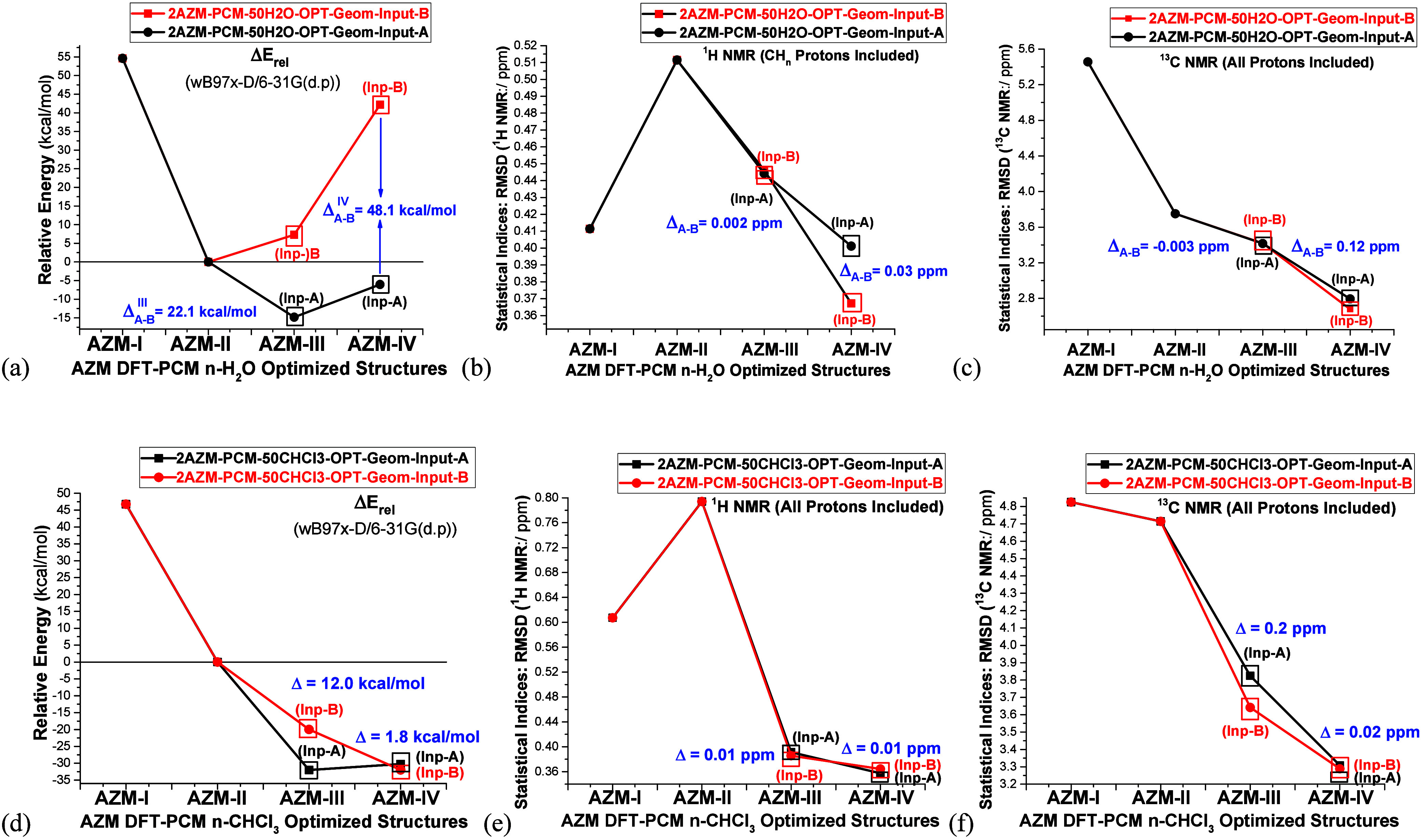
(a–c) DFT-PCM-50CHCl_3_ and (d–f) DFT-PCM-50H_2_O relative energies (kcal mol^–1^) and RMSD NMR values (ppm) for relevant AZM structures, using two distinct manually modified inputs of structures for **AZM-III** and **AZM-IV** in the geometry optimization procedure, named Input-A and Input-B.

Relative energy and RMSD results (Input-A and Input-B) for water and chloroform are given in Figure 11a–c and 11d–f, respectively. The dependence of relative values on the initial guess structure in water solution is like that of DMSO, with the **2AZM-IV-50H**_**2**_**O** Input-B structure being destabilized with respect to Input-A by a large amount of approximately 48 kcal mol^–1^. Regarding the RMSD value, practically the same results are obtained with both Input-A and Input-B, with maximum deviations (Δ_A-B_^III^ and Δ_A-B_^IV^) of 0.03 and 0.12 ppm observed respectively for ^1^H and ^13^C NMR chemical shifts, similar to DMSO, showing also a very low sensitivity to the initial guess geometry.

For chloroform solvent the change in relative energy is small for the **2AZM-IV** structure (Δ_A-B_^IV^ = 1.8 kcal mol^–1^) and larger for the **2AZM-III** dimer (Δ_A-B_^III^ = 12.0 kcal mol^–1^) but smaller than water (22.1 kcal mol^–1^). RMSD values are almost insensitive to the initial guess structures (Input-A and Input-B) with the largest deviations being 0.01 and 0.2 ppm (^1^H and ^13^C NMR values, respectively), following the same behavior observed in DMSO and water solutions.

Table 2 reports intra- and intermolecular H-bond distances (in Å) for the **AZM-IV** dimeric structure (PCM-50CHCl_3_, PCM-50DMSO, and PCM-50H_2_O species), which is the most probable to exist in solution according to the NMR predictions. It can be seen from Table 2 that the intramolecular H-bond distances and shortest solute–solvent distances are very similar for the **AZM-IV** dimeric structures optimized using two distinct guest inputs (A and B) for all three solvents. This aligns with the RMSD NMR patterns shown in Figures 8 and 10. Most importantly, it strongly indicates that the notable change in the relative energies of structures **AZM-III** and **AZM-IV** is specifically due to solvent–solvent interactions, which significantly affect the calculated DFT total energies of each structure. The results reported in Figures 9 and 11 revealed that for highly solvated structures a degree of arbitrariness in the calculation of relative energies is naturally introduced, mainly due to solvent–solvent interaction causing a strong dependence of total energies on the initial guess structure used in the geometry optimization procedure, and therefore, it may not be used as a criterion to find the predominant conformer in solution, since any new DFT optimized explicitly solvated AZM structure may be obtained when starting the geometry optimization procedure with a new input guess geometry. This does not happen with NMR statistical indices results. As already mentioned, NMR chemical shifts are not greatly influenced by changes in solvent–solvent interactions far away from the solute; it depends mainly on the local chemical environment around the solute. Therefore, analysis of NMR data seems more adequate than DFT-PCM calculated relative energies in conformational analysis of organic molecules.

**Table 2:** ωB97x-D/6-31G(d,p) PCM-50CHCl_3_, PCM-50DMSO, and PCM-50H_2_O Intramolecular O–H···ωO and O–H···N Distances (Å) and AZM···CHCl_3_, AZM···DMSO, and AZM···H_2_O Shortest Intermolecular OH Groups Distances (Å) for **AZM-IV** Dimeric Structures

	**Intramolecular Distances (Å)**				
**DFT-PCM Optimization**	**C2′–OH···N**	**C4′′–OH···O**	**C6-OH···N**	**C11-OH···O=C**	**C12-OH···O–H**
**2AZM-50CHCl**_**3**_**-Input-A**	2.25	2.29	1.69	1.90	2.10
**2AZM-50CHCl**_**3**_**-Input-B**	2.33	2.28	1.68	1.91	2.11
**2AZM-50DMSO-Input-A**	2.47	2.21	1.63	3.74	2.16
**2AZM-50DMSO-Input-B**	2.42	2.23	1.63	3.62	2.24
**2AZM-50H**_**2**_**O-Input-A**	2.46	2.36	1.67	2.28	2.22
**2AZM-50H**_**2**_**O-Input-B**	2.44	2.37	1.71	2.46	2.2

	**Intermolecular Shortest Distances (Å)**				
	**AZM···CHCl**_**3**_				
**DFT-PCM Optimization**	**C2′–OH···Cl**	**C4′′–OH···Cl**	**C6-OH···HCCl**_**3**_	**C11-OH···Cl**	**C12-OH···Cl**
**2AZM-50CHCl**_**3**_-Input-A	2.70	2.31	1.96	2.91	2.65
**2AZM-50CHCl**_**3**_-Input-B	2.73	2.31	2.00	2.95	2.64

	**AZM···DMSO**				
	**C2′–OH···O = S**	**C4′′–OH···O = S**	**C6–O···S = O**	**C11-OH···O = S**	**C12-OH···O = S**
**2AZM-50DMSO-**Input-A	1.84	1.98	3.23	1.72	1.97
**2AZM-50DMSO-**Input-B	1.84	1.99	3.14	2.40	1.90

	**AZM···H**_**2**_**O**				
	**C2′–OH···O**	**C4′′–OH···O**	**C6-OH···HOH**	**C11-OH···O**	**C12-OH···O**
**2AZM-50H**_**2**_**O-**Input-A	1.77	1.68	1.67	1.86	1.81
**2AZM-50H**_**2**_**O-**Input-B	1.745	1.69	1.74	1.82	1.82

Solvent–solvent interactions are indeed misguided and should be eliminated. One way to address this issue is by performing fragment calculations on the final structures of the investigated systems and then analyzing the energy of the solute alone. To achieve this, we conducted single-point DFT energy calculations on the supermolecules but eliminated the solvent molecules from the total energy calculations. This method maintained the influence of explicit solvent effects on determining the optimized molecular structure of AZM, while focusing solely on the energy analysis of the solute. Therefore, we excluded the extra energy terms for both solvent–solvent and solute–solvent interactions. and the results are presented as Supporting Information (Figure S4). It can be seen that the effect for water and DMSO polar solvents is remarkable both for energy trend and magnitude of relative energies, predicting the **AZM-IV** structure as favorable over **AZM-III** in both solvents by approximately 3 and 4 kcal mol^–1^, respectively. The situation is reversed in chloroform by 2 kcal mol^–1^, which can be considered energy degenerate. Such an approximate procedure can be an alternative to eliminate the solvent–solvent interactions from the evaluation of relative energies of different structures of the same molecule. This is, indeed, a delicate theoretical issue.

Regarding comparison with experimental NMR spectra, the use of Boltzmann averaged computed NMR chemical shifts for the four AZM structures could be an adequate procedure, once the theoretical NMR profiles do not match nicely the observed experimental pattern. Experimental measurement offers signals averaged over existing low-energy conformers. However, it would be dependent on the relative energy values, which are highly influenced by solvent–solvent interactions, as discussed above. This procedure would be fine without the inclusion of explicit solvent molecules in the relative energy calculations. We have partially done that for structures **AZM-III** and **AZM-IV**, assuming an equal Boltzmann weight just to see the effect on the NMR spectrum. The results are shown in Figures S1n,o and S2n,o (Supporting Information), and no considerable improvement in the agreement with experimental NMR profile was observed. In addition, looking at the relative energy profiles reported in Figures 2a, 6a, and 7a, energy differences have appreciable values not leading to a Boltzmann population for the four conformers, which would produce a sizable effect on the DFT calculated average NMR spectra.

**Figure 12:**
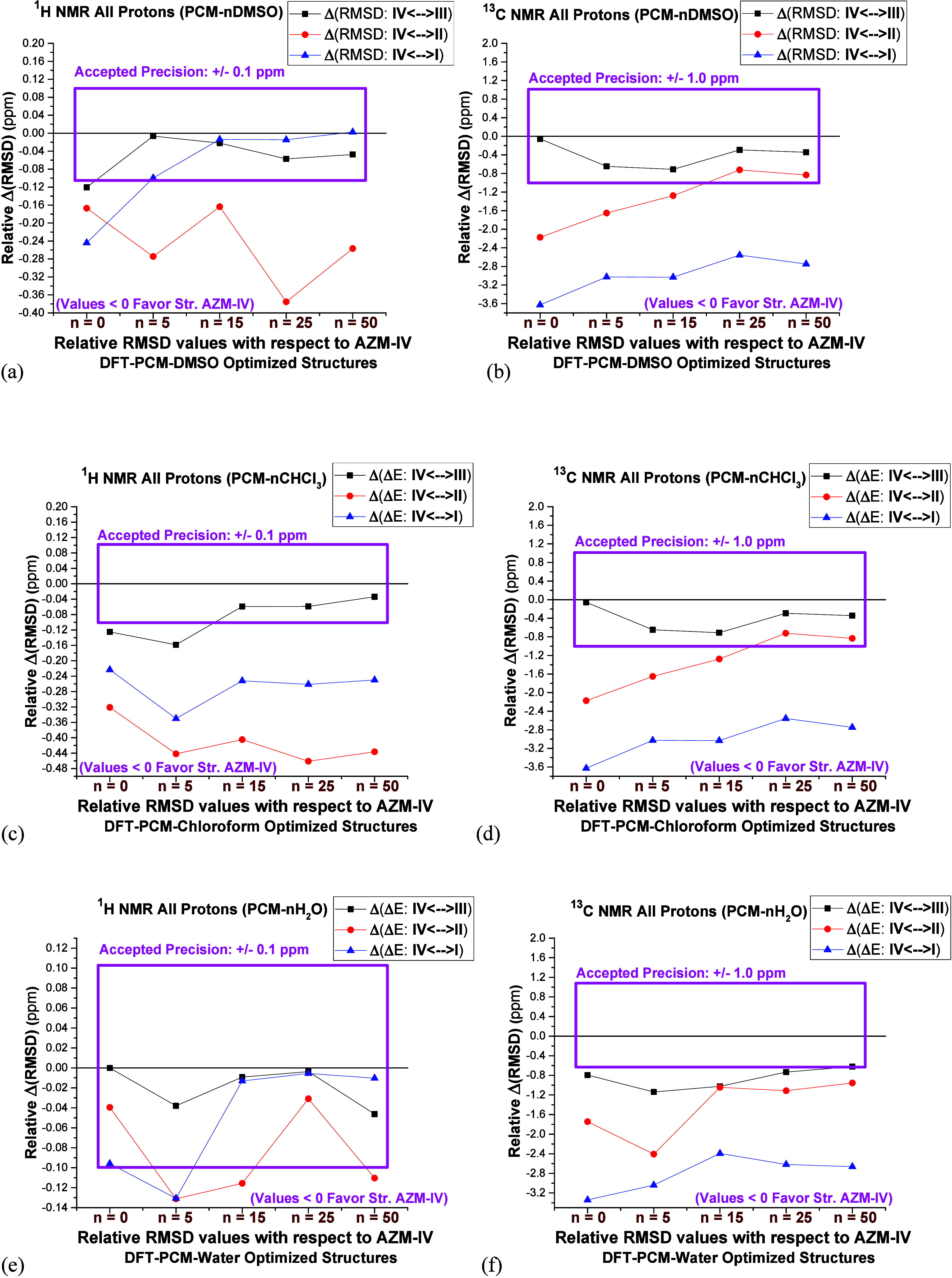
(a, b) PCM-*n*DMSO, (c, d) DFT-PCM-*n*CHCl_3_, and (e, f) PCM-*n*H_2_O (*n* = 0, 5, 15, 25, and 50) relative RMSD NMR values (Δ(RMSD) in ppm) for structure **AZM-IV** with respect to **AZM-I**, **AZM-II**, and **AZM-III**. For Δ(RMSD) values less than zero (<0) the **AZM-IV** structure is predominant. This is indicated in Figure 11. A relative RMSD values uncertainty window, representing the commonly accepted standard precision of energy ^1^H NMR (0.1 ppm) and ^13^C NMR (1 ppm) data, is shown in a pink rectangle. For relative NMR data inside the window, all conformers of azithromycin are equally probable to exist in the solution. (a, b) PCM-*n*DMSO, (c, d) PCM-*n*CHCl_3_, and (e, f) PCM-*n*H_2_O.

Finally, when comparison with experimental data is made to reach a conclusion (which is a straightforward procedure in chemistry), a point that promptly emerges is how comfortable is a deviation from experimental values (Δ*E* or NMR) between two plausible molecular structures (**AZM-III** and **AZM-IV** in this study). More specifically, among various structures of the same molecule, how small should the RMSD and Δ*E* deviation values be to confidently determine the predominant conformer of a given organic molecule? Although the DFT-PCM RMSD NMR patterns shown previously using chloroform (Figure 2), DMSO (Figure 6), and water (Figure 7) solvents allowed us, in principle, to predict the predominance of the **AZM-IV** structure in solution, the theoretical results should be analyzed in light of the expected uncertainty of the DFT methodology. As mentioned before,^25,29^ DFT methods can predict ^1^H and ^13^C NMR chemical shifts with a high accuracy of 0.1 and 1 ppm, respectively. Regarding energy values, DFT method accuracies are limited to 2–3 kcal mol^–1^, close to the quantum chemical accuracy (∼1 kcal mol^–1^).^30^ These are the RMSD NMR chemical shift threshold value windows shown in Figure 12. It should be mentioned that the deviation (Δ(RMSD)) given in Figures 9 and 11 is well below 0.1 ppm (^1^H NMR) and 1 ppm (^13^C NMR), assuring that NMR chemical shifts for Inputs A and B of the AZM solvated dimer structure are truly the same.

It can be seen from Figure 12a,b that ^1^H NMR and ^13^C NMR data allow us to differentiate between structures **AZM-IV** and **AZM-II** and **AZM-IV** and **AZM-I**, respectively, when 50 explicit DMSO solvent molecules are included. Structures **AZM-III** and **AZM-IV** are both likely to exist in DMSO solution and cannot be identified, which is also observed in chloroform (Figure 12c,d) and water (Figure 12e,f) solution. Using ^13^C NMR PCM-*n*H_2_O results, **AZM-IV** and **AZM-II** and **AZM-IV** and **AZM-I** can be separated. Analysis of ^1^H NMR relative RMSD (PCM-50H_2_O) is less clear, and no predominance of a specific AZM structure can be predicted (Figure 12e). The patterns shown in Figure 12 can be used in other analyses of NMR data for organic molecules, aiming at the determination of preferred conformers in solution among various possible structures with a degree of confidence. It provides us with an adequate procedure for using NMR data in conformational analysis, where only the data outside the threshold precision window can be trusted. Analyzing the deviation between experimental and DFT-PCM NMR chemical shift values for a series of candidate molecular structures is a common procedure in chemistry for elucidating preferred conformations, making the data reported in Figure 12 particularly relevant.

**Figure 13:**
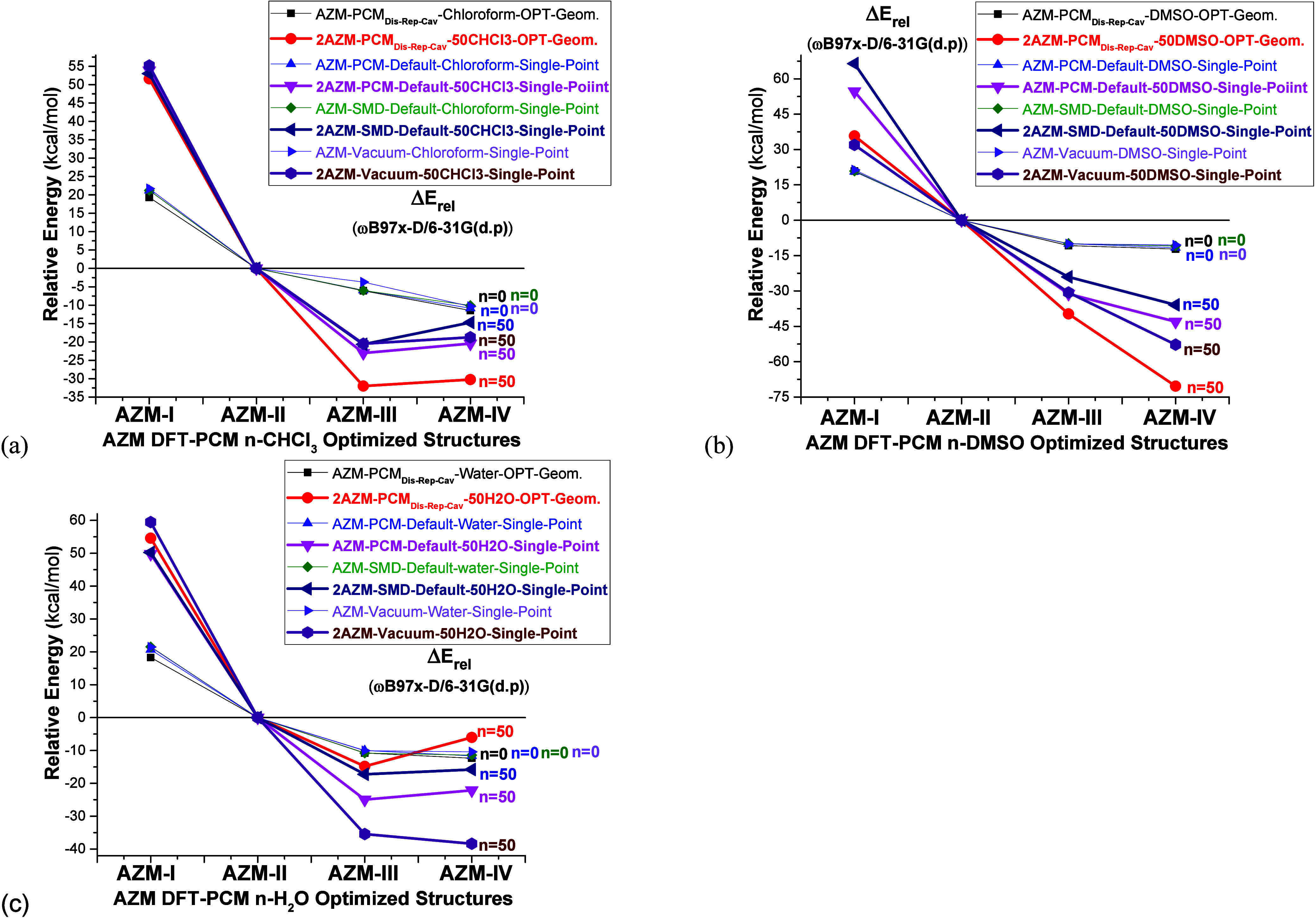
ωB97x-D/6-31G(d,p) (a) PCM-*n*CHCl_3_, (b) PCM-*n*DMSO, and (c) PCM-*n*H_2_O (*n* = 0, 50) relative energies for AZM structures, using different solvation models.

Lastly, the effect of choosing another well-known continuum solvation model (SMD)^2^ and the use of explicit solvent molecules in DFT calculations in the vacuum were analyzed. The results are presented in Figure 13. Two PCM results are included related to the presence or absence of the dispersion, repulsion, and cavity contributions to the total energy: PCM_**Dis-Rep-Cav**_ (presence) and PCM-Default (absence). The PCM_**Dis-Rep-Cav**_ was called so far simply as “PCM”. When an implicit solvent model is used for geometry optimization, all four single-point relative energy calculations converge to the same result. For DFT optimized solvated dimer structures, the SMD, PCM-default, and vacuum results follow virtually the same trend leading to similar predictions in chloroform and DMSO solution, with a higher stabilization of the **AZM-IV** (and **AZM-III**) solvated dimer structures observed at the PCM_**Dis-Rep-Cav**_ level compared to the other three levels, but keeping the same energy trend. The vacuum results, i.e., including explicit solvent molecules without using the continuum model, follow the same trend as PCM-default and SMD, indicating that the use of a continuum model in calculations of explicitly solvated structures plays a minor role in the energy profile; however, the PCM_**Dis-Rep-Cav**_ model improves substantially the stabilization of **AZM-III** and **AZM-IV** with respect to the **AZM-II** structure for chloroform and DMSO solvents. The PCM_**Dis-Rep-Cav**_ relative energy results for **AZM-III** and **AZM-IV** solvated dimers in water solution are rather different from DMSO, being destabilized with respect to SMD, PCM-default, and vacuum levels of calculation. This is a behavior that deserves further attention.

## Conclusions

In this article, we present a discussion about the interpretation and determination of theoretical data, especially when comparing experimental and theoretical results (a common procedure in chemistry) to draw conclusions about a chemical process or conformational analysis. A key point that quickly arises is how small must the deviation from experimental data (RMSD for NMR chemical shifts) be for theoretical predictions to be considered reliable? This is a crucial factor in determining the accuracy of such predictions. Where ^1^H NMR chemical shift is concerned, deviations (ΔRMSD between two species) lower than 0.1 ppm can be considered small enough to establish the coexistence of distinct structures. Regarding energy differences between two given structures, calculated values in the range of 1–2 kcal mol^–1^ would also be sufficient to characterize degenerate conformers of the same molecule. But most of the time this criterion does not exactly match, and we have intermediate deviations among various possible structures of a given molecule. We may use a percentage deviation to ease comparison. In this work we used as a working example the antibiotic azithromycin (AZM), which is a large and flexible organic compound, containing five OH groups and other polar centers susceptible to interaction with solvent molecules, being a representative molecule for investigating solvent effects on conformational analysis. There are four distinct azithromycin structures (**AZM-I**, **AZM-II**, **AZM-III**, and **AZM-IV**) that can be used as good examples of how deviation from chemical shift experimental data (RMSD and NMR spectra) and relative energies can guide us to the determination of the predominant structure in solution. This can be extended to conformational analysis of other large organic molecules. The structural difference between **AZM-III** and **AZM-IV** is essentially the H-bond involving the O–H2′ group, O–H2′···O and O–H2′···N, respectively (Figure 1c,d). A comparative analysis of experimental and theoretical NMR spectra, calculated using DFT-PCM with the inclusion of explicit solvent molecules, can be considered a useful approach in structural elucidation. Since thermodynamic data in solution are typically unavailable for comparison, we must rely on the size of DFT-PCM calculated Δ*E* values for a series of candidate molecular structures, with energy differences close to 1–2 kcal mol^–1^ implying degenerate conformers.

It is interesting to analyze how the specific solvation model used can affect each structure differently, which is revealed in the results reported for chloroform, DMSO, and water solvents. For the low polar solvent chloroform, the implicit solvent (*n* = 0) and PCM-25CHCl_3_ model predicted structure **IV** as predominant by 5 kcal mol^–1^, with the 2AZM-PCM-50CHCl_3_ relative energy giving equal probability for structures **III** and **IV**. Structure **III** is favored at the 3AZM-PCM-75CHCl_3_ level by 7 kcal mol^–1^. These results illustrate the sensitivity of Δ*E*_rel_ values to the model of solvation. All RMSD NMR chemical shift trends (PCM-*n*CHCl_3_) predicted structure **IV** as the preferred one, with the lowest statistical indices found when implicit solvation or only five explicit solvent molecules are used. The RMSD ^1^H NMR data when 15, 25, 50, and 75 CHCl_3_ solvent molecules are included are virtually the same, predicting both structures (**III** and **IV**) to exist in solution. However, the RMSD ^13^C NMR trend is the same no matter what solvation model is used, giving a clear predominance for structure **AZM-IV**.

In DMSO and water, the energy predictions are opposite regarding the preference for structure **AZM-III** or **AZM-IV**. The RMSD results for NMR chemical shifts strongly indicated structure **AZM-IV** as preferred in DMSO, such as in the chloroform case. The same conclusion is obtained in a water solution regarding ^13^C NMR results. However, as discussed before, the RMSD values reported here should be looked at in light of the expected precision of DFT based methods for the calculation of ^1^H NMR (0.1 ppm) and ^13^C NMR (1 ppm) chemical shifts. Therefore, the DFT PCM calculated NMR data reported for chloroform, DMSO, and water solvents cannot discriminate between structures **AZM-III** and **AZM-IV**, since the difference in the respective RMSD values is within a threshold limit value. So, they are both probable to exist in solution based on NMR analysis.

Our results strongly indicate that finding a global minimum among many possible local minima present on the PES for highly solvated AZM structures is a desired but not a feasible computational task. As the number of explicit solvent molecules around the solute increases, the degrees of freedom rise enormously, and jumping to the lowest-energy structure during DFT-PCM geometry optimization is not so straightforward. A local minimum-energy structure, like the initial guess, is always predicted due to the presence of many structurally close stationary points on the potential energy surface (PES). This prediction is strongly influenced by numerous attractive solvent–solvent interactions, which lower the total energy without significantly altering the solute’s geometry, while still meeting the convergence criteria for geometry optimization. This does not happen when only five explicit solvent molecules are placed strategically around each OH group of AZM, and in this case, precise molecular geometry is unambiguously determined. The collective solvent effect (solute–solvent and solvent–solvent interactions) on the total energies makes a quantum chemical investigation of the preferred molecular structure in solution for large, solute-solvated clusters.

In this sense, the use of continuum models including a few solvent molecules strategically placed around the solute (five in the case of AZM) to describe solvent effects, using our chemical intuition, seems more feasible from a computational point of view. It worked fine in the case of the low polar chloroform solvent, yielding similar relative energies and RMSD NMR trends as the PCM-*n*CHCl_3_ solvation model (*n* = 50). The same holds true for water and DMSO solvents. In the case of these polar solvents, increasing the number of explicit solvent molecules (*n* > 5) causes a larger deviation from the implicit solvation model results. Our results revealed that while RMSD NMR patterns are moderately affected by increasing the number of explicit solvent molecules, the relative energy profiles may be remarkably influenced by the solvation model used and may not be quite trusted since solvent–solvent interactions may cause large changes in the total energies of distinct conformers leading to an unrealistic preference for a given molecular structure based on energetic grounds.

Our theoretical results allow us to make a critical analysis of the inclusion of explicit solvent molecules in DFT geometry optimization and NMR calculations of organic molecules. These are the main questions to be asked: (i) Is it necessary to use a highly solvated molecular system for the prediction of relative energies of distinct conformers of the same molecules and NMR chemical shifts? (ii) Are great computational efforts justified? (iii) Is the implicit solvent model enough? (iv) What is the best strategy, using computational quantum chemistry, for the elucidation of the predominant structure in solution? The results reported here enable us to address these points. For low polar solvents, such as chloroform, the PCM continuum model or including only five explicit CHCl_3_ molecules leads essentially to the same trend predictions as larger calculations for dimer (2AZM-50CHCl_3_) and trimer (3AZM-75CHCl_3_) structures. However, for the polar DMSO and water solvents, relative energies are significantly affected by increasing the number of solvent molecules around the solute, but NMR statistical index trends are roughly the same and great computational efforts may not be necessary for the evaluation of NMR statistical indexes, with the inclusion of only five solvent molecules around OH groups being sufficient, in the case the AZM molecule. In addition, our results strongly indicated that for evaluation of ^13^C NMR chemical shifts the PCM model alone is adequate. Regarding the best strategy for structural elucidation in solution, the relative energy criterion does not seem very reliable, when many explicit solvent molecules are included in DFT calculations, and in this case, we should rely on the analysis of DFT-PCM calculated NMR chemical shift data, which can be directly compared with experimental data. Even so, deviations among distinct conformers within a threshold limit value for ^1^H NMR and ^13^C NMR (0.1 and 1.0 ppm, respectively) leave such structures equally probable to exist in solution (two structures, **AZM-III** and **AZM-IV**, in the case of azithromycin).

## Supporting Information Available

Additional figures and tables, experimental section, and optimized Cartesian coordinates of all structures shown in this article (PDF)
