# Supplementary material for: Modeling Solvent Effects in Quantum Chemical Calculation of Relative Energies and NMR Chemical Shifts for Azithromycin
Source: J Phys Chem A. 2025 Feb 22;129(9):2200–16. doi: 10.1021/acs.jpca.4c08015 (PMC11891909; doi:10.1021/acs.jpca.4c08015)
Supplement: Supplementary file 1 — jp4c08015_si_001.pdf [file jp4c08015_si_001.pdf]

SUPPLEMENTARY MATERIAL

MODELLING SOLVENT EFFECTS IN QUANTUM CHEMICAL CALCULATION OF RELATIVE ENERGIES AND NMR CHEMICAL SHIFTS FOR ORGANIC MOLECULES

Haroldo C. Da Silva[a], Isabel S. Hernandes[a], Wagner B. De Almeida[a,*]
[a] Laboratório de Química Computacional e Modelagem Molecular (LQC-MM), Departamento de Química Inorgânica, Instituto de Química, Universidade Federal Fluminense (UFF), Outeiro de São João Batista s/n, Campus do Valonguinho, 24020-141, Centro, Niterói, RJ, Brazil.
[b] Departamento de Físico-Química, Instituto de Química, Pavilhão Haroldo Lisboa da Cunha, Universidade do Estado do Rio de Janeiro (UERJ), Rua São Francisco Xavier, 524, 20550-013, Maracanã, Rio de Janeiro, RJ, Brazil.

Table S1. X-ray and DFT-optimized torsion angles (º) for the AZM structures are shown in Figure 1.

	**ωB97x-D/6-31G(d,p) Vacuum Optimized Structure**				**Solid-State**
	**AZM-I**	**AZM-II**	**AZM-III**	**AZM-IV**	**X-Ray**
	**Macrocyclic Ring**				
φ_1 : [C1-C2-C3-C4]	-62.3	-59.8	-113.1	-87.8	-89.9
φ_2: [C2-C3-C4-C5]	81.4	144.2	163.3	164.9	177.2
φ_3 : [C3-C4-C5-C6]	-118.4	167.7	-101.7	-103.2	-108.7
φ_4 : [C4-C5-C6-C7]	-80.6	56.6	-71.1	-75.2	-71.9
φ_5 : [C5-C6-C7-C8]	143.3	82.9	-177.9	-177.7	173.6
φ_6 : [C6-C7-C8-C9]	45.8	-129.4	-88.9	-95.8	-106.0
φ_7 : [C7-C8-C9-N]	-88.8	-29.6	62.0	60.2	64.7
φ_8 : [C8-C9-N-C10]	157.0	164.7	-152.0	-149.7	-147.6
φ_9 : [C9-N-C10-C11]	-169.2	-67.9	137.1	140.9	158.5
φ_{10} : [N-C10-C11-C12]	38.2	-150.8	-173.2	-167.5	-155.8
φ_{11} : [C10-C11-C12-C13]	61.6	174.8	170.3	170.6	161.4
φ_{12} : [C11-C12-C13-O]	-152.1	-60.7	-60.1	-58.7	-76.3
φ_{13} : [C12-C13-O-C1]	150.2	99.6	118.9	101.1	118.8
φ_{14} : [C13-O-C1-C2]	-162.5	170.8	-174.8	172.3	176.9
φ_{15} : [O-C1-C2-C3]	148.5	147.5	130.8	135.3	121.9
φ_{16} : [CH_3-CH2-C13-O]	178.1	70.4	67.1	68.8	70.4
	**Sugar Units**				
φ_{17} : [O-C5-C4-C3]	119.0	41.4	133.7	133.8	129.7
φ_{18}: [C1'-O-C5-C4]	-102.4	-146.1	-109.1	-105.9	-101.5
φ_{19} : [C2'-C1'-O-C5]	-70.6	134.0	159.8	159.0	156.3
φ_{20} : [C3'-C2'-C1'-O]	176.9	174.0	178.6	173.7	179.6
φ_{21} : [C4'-C3'-C2'-C1']	-54.4	-53.7	-56.9	-57.8	-58.5
φ_{22} : [C5'-C4'-C3'-C2']	55.3	53.0	55.7	56.9	55.2
φ_{23} : [O-C5'-C4'-C3']	-57.3	-56.1	-54.4	-54.8	-53.5
φ_{24} : [O-C3-C4-C5]	-161.3	-99.8	-72.4	-72.0	-59.4
φ_{25}: [C1''-O-C3-C4]	106.8	111.6	151.1	146.9	145.2
φ_{26} : [C2''-C1''-O-C3]	-155.2	-157.8	158.9	159.0	152.5
φ_{27} : [C3''-C2''-C1''-O]	-175.9	-179.4	76.6	78.6	76.6
φ_{28} : [C4''-C3''-C2''-C1'']	53.4	51.3	50.3	50.7	49.4
φ_{29} : [C5''-C4''-C3''-C2'']	-50.5	-47.9	-53.2	-53.7	-52.2
φ_{30} : [O-C5''-C4''-C3'']	52.5	53.1	56.0	55.7	56.0
φ_{31} : [CH_3-N-C3'-C2']	76.7	77.1	67.1	77.2	48.8
**φ_{32} : [CH_3-N-C3'-C2']**	**-154.9**	**-153.9**	**-162.4**	**-154.2**	**-80.5**
φ_{33} : [CH_3-O-C3''-C2'']	-57.6	-53.9	-57.9	-57.2	-52.7

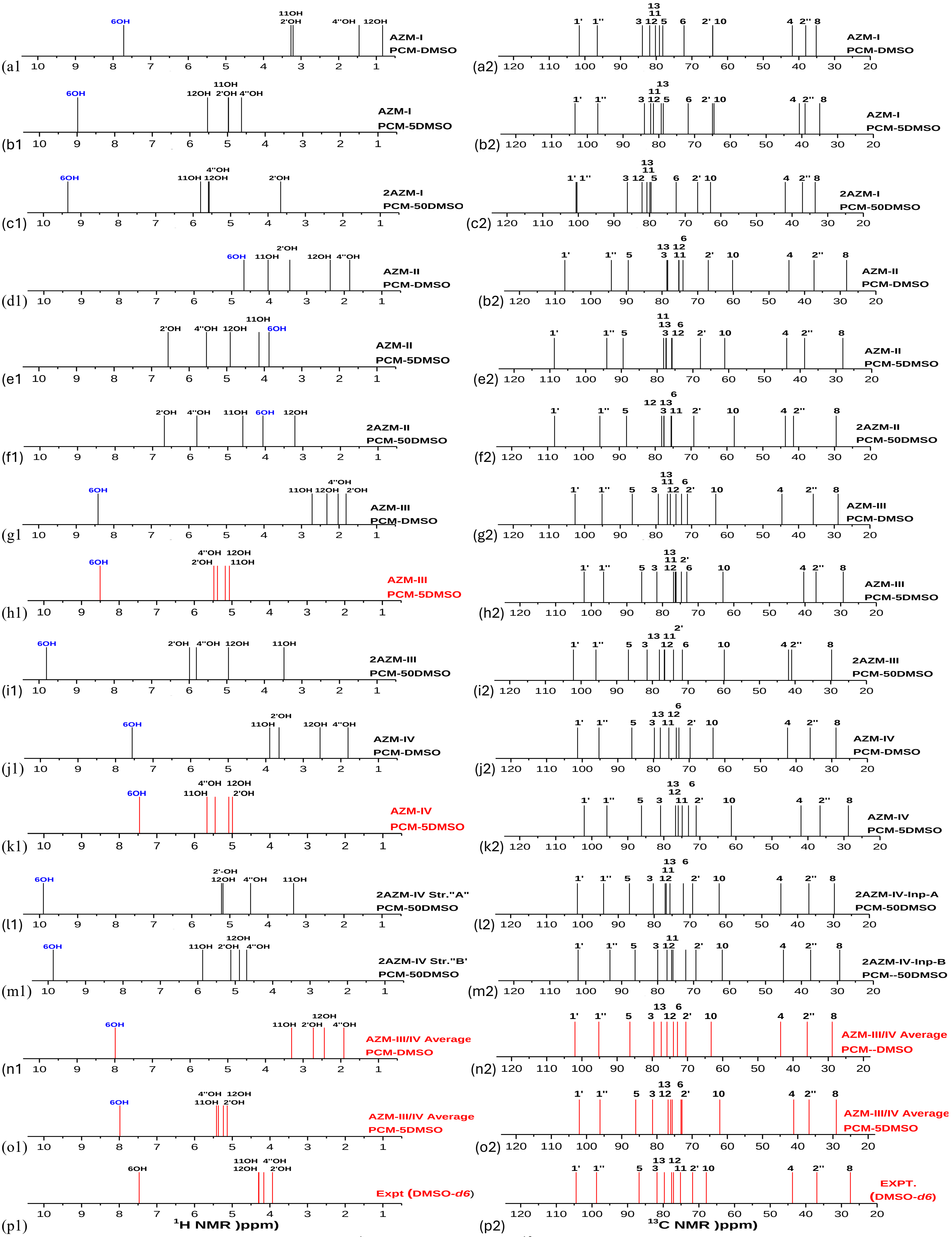

Figure S1. B3LYP/6-31G(d,p)-PCM-nDMSO ^{1}H (OH protons) and ^{13}C NMR spectra for AZM solvated structures.

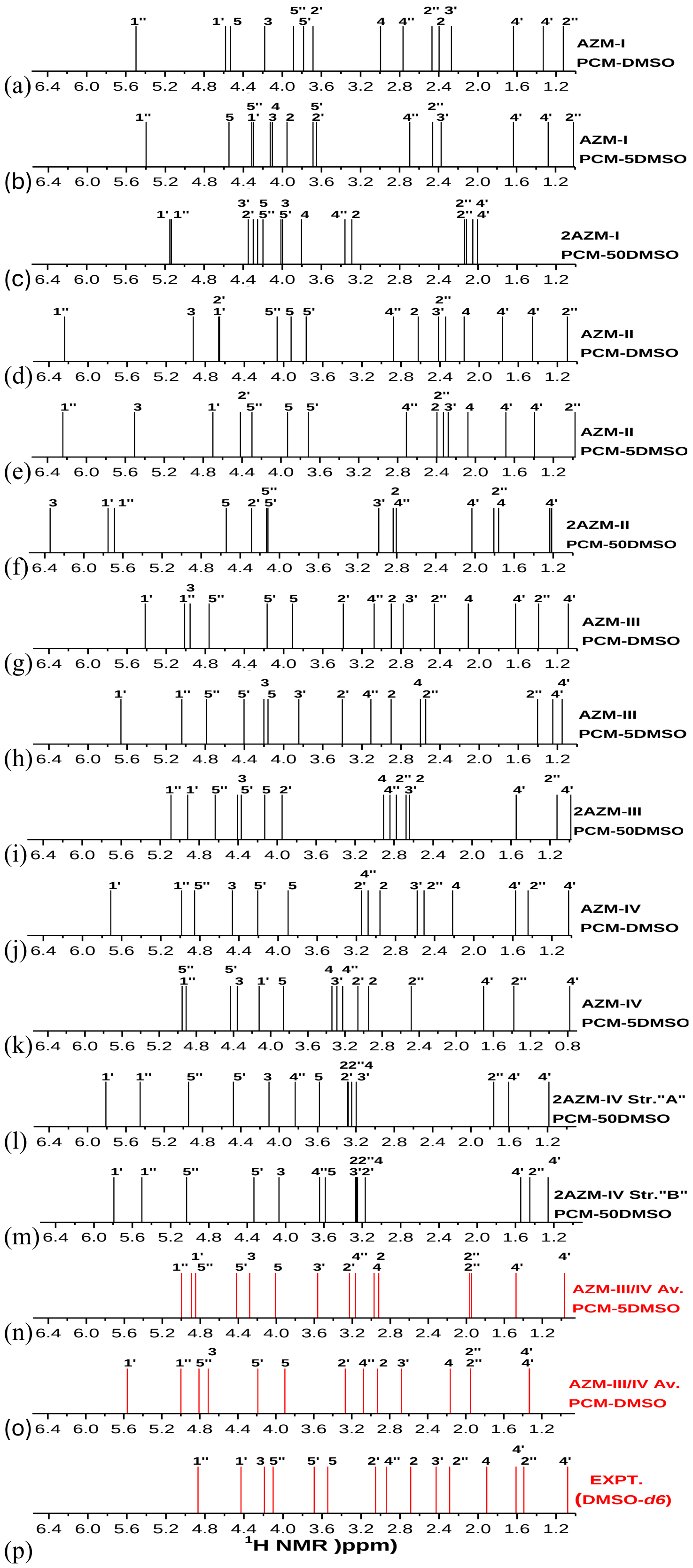

Figure S2. B3LYP/6-31G(d,p)-PCM-nDMSO ^{1}H NMR spectra (CH_n protons) for AZM solvated structures (n = 0; n = 50)

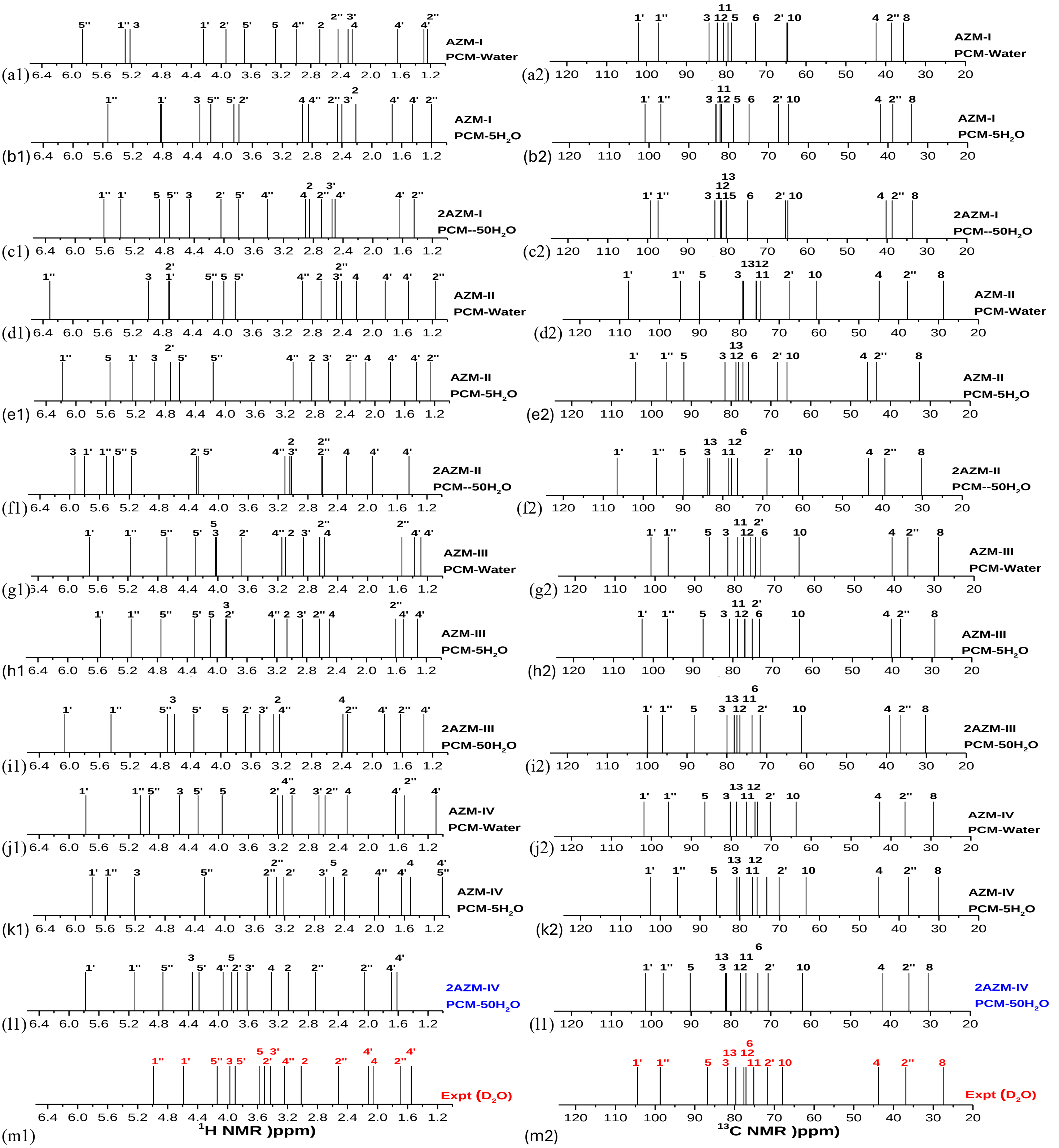

Figure S3. B3LYP/6-31G(d,p)-PCM-nH_2O ^{1}H NMR and ^{13}C NMR spectra for AZM solvated structures (n = 0 and n = 50) (a-l) and experimental spectrum (in D_2O) (m). Selected protons were used for easy spectrum visualization.

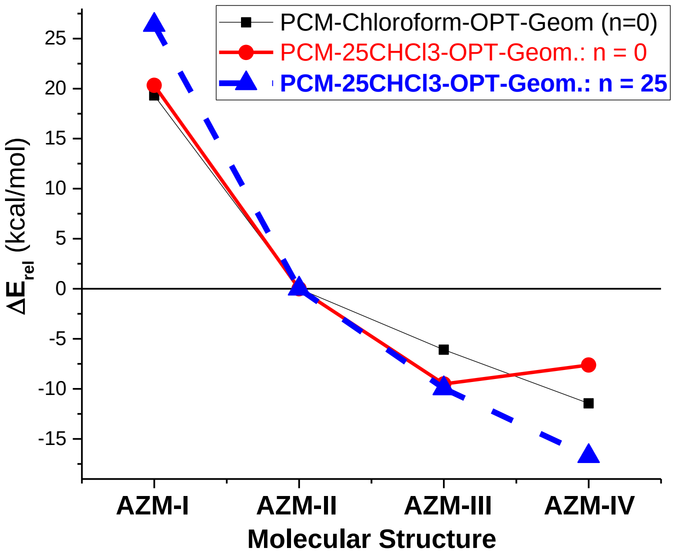

(a)

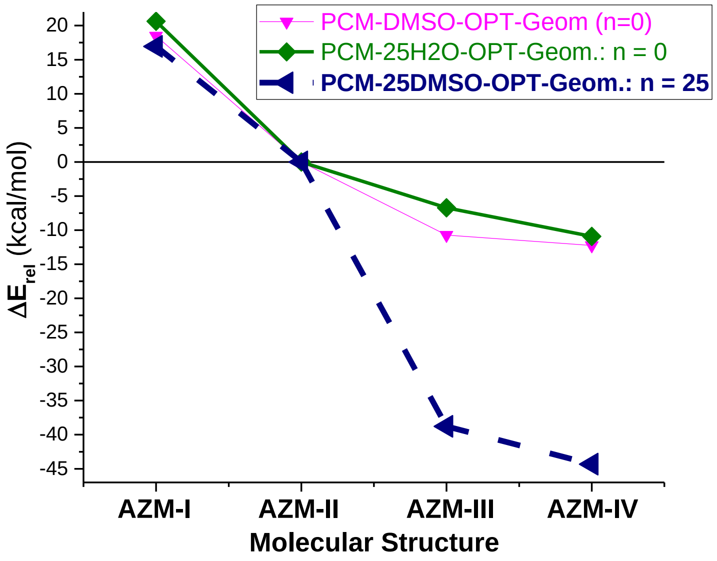

(b)

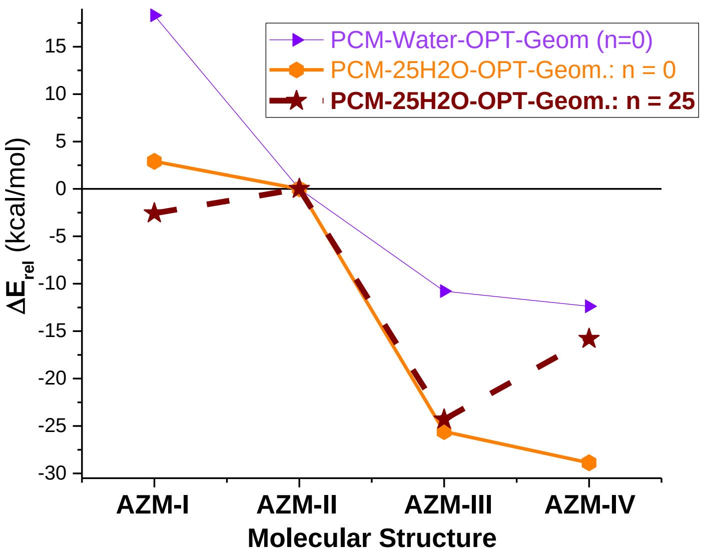

(c)

Figure S4. ωB97x-D/6-31G(d,p) (a) PCM-n$CHCl_3$, (b) PCM-nDMSO, and (c) PCM-nH_2O (n = 0, 25) relative energies for AZM structures, using an approximate procedure to eliminate the solvent-solvent interactions contribution to the total energies. The total energy of each solvated structure (n = 25) is evaluated using the respective optimized solvated geometry but deleting all 25 solvent molecules, like a PCM implicit model approach (named PCM-25$CHCl_3$-OPT-Geom.: n = 0, for chloroform). Therefore, the solvent effect is considered only on the solute molecular structure, not on the calculated total energy where the continuum model is employed.
